# Light from dark: A relictual troglobite reveals a broader ancestral distribution for kimulid harvestmen (Opiliones: Laniatores: Kimulidae) in South America

**DOI:** 10.1371/journal.pone.0187919

**Published:** 2017-11-30

**Authors:** Abel Pérez-González, F. Sara Ceccarelli, Bruno G. O. Monte, Daniel N. Proud, Márcio Bernardino DaSilva, Maria E. Bichuette

**Affiliations:** 1 División Aracnología, Museo Argentino de Ciencias Naturales "Bernardino Rivadavia"—CONICET, Buenos Aires, Argentina; 2 Programa de Pós-Graduação em Ecologia e Recursos Naturais, Universidade Federal de São Carlos, Rodovia Washington Luis, São Carlos, Brasil; 3 Departamento de Ecologia e Biologia Evolutiva, Universidade Federal de São Carlos, Rodovia Washington Luis, São Carlos, Brasil; 4 Departamento de Sistemática e Ecologia, Universidade Federal da Paraíba, Paraíba, Brasil; Indiana University Bloomington, UNITED STATES

## Abstract

A new troglobitic harvestman, *Relictopiolus galadriel* **gen. nov** *et* **sp. nov.**, is described from Olhos d’Água cave, Itacarambi, Minas Gerais State, Brazil. Morphological characters, including male genitalia and exomorphology, suggest that this species belongs to the family Kimulidae, and it appears to share the greatest similarities with *Tegipiolus pachypus*. Bayesian inference analyses of a molecular dataset strongly support the inclusion of this species in Kimulidae and confirm the hypothesized sister-group relationship between *R*. *galadriel* and *T*. *pachypus*. A time calibrated phylogeny indicates that these sister-taxa diverged from a common ancestor approximately 40 Mya, during the Paleogene. The current range of Kimulidae illustrates a remarkable disjunct distribution, and leads us to hypothesize that the ancestral distribution of Kimulidae was once much more widespread across eastern Brazil. This may be attributed to the Eocene radiation associated with the warming (and humidifying) events in the Cenozoic when the best conditions for evergreen tropical vegetation in South America were established and followed by the extinction of kimulid epigean populations together with the retraction of rain forests during the Oligocene to Miocene cooling. The discovery of this relictual troglobite indicates that the Olhos d’Água cave was a stable refugium for this ancient lineage of kimulids and acted as a "museum" of biodiversity. Our findings, considered collectively with the diverse troglofauna of the Olhos d’Água cave, highlight it as one of the most important hotspots of troglobite diversity and endemism in the Neotropics. Given the ecological stresses on this habitat, the cavernicolous fauna are at risk of extinction and we emphasize the urgent need for appropriate conservation actions. Finally, we propose the transfer of *Acanthominua*, *Euminua*, *Euminuoides* and *Pseudominua* from Kimulidae to Zalmoxidae, resulting in two new synonymies and 13 new combinations.

## Introduction

Morphological forms that are derived via adaptations to dark subterranean environments display a high degree of evolutionary convergence. Across diverse animal taxa, from fish and salamanders to arachnids and myriapods, troglomorphism is characterized by the loss of pigments and eyes, the elongation of appendages, and the elaboration of extra-optic sensory structures [1]. It is therefore no surprise that troglobites—species with life cycles exclusively in caves—have instilled a sense of awe and bewilderment in taxonomists and evolutionary biologists for more than three centuries. Through the lens of a taxonomist, a troglobite represents an exquisite, seemingly bizarre taxon, incredibly divergent from even their closest epigean relatives, thus making them relatively easy to diagnose while simultaneously presenting a challenge to hypothesizing interspecific relationships. Meanwhile, through the lens of the evolutionary biologist, caves represent important laboratories of evolution [1,2] and cave faunas offer exceptional biological models to study the underlying evolutionary processes that drive adaptation and speciation to produce such remarkably convergent forms.

Subterranean ecosystems have a unique combination of features including: i) truncated food webs, ii) relatively few lineages, iii) a high proportion of endemic species and many allopatric vicariant species, and iv) high level of relictual taxa [3]. The latter two features make troglobitic taxa ideal candidates for phylogenetic and biogeographic reconstructions. In the arachnid order Opiliones, known as harvestmen, there is a considerable number of relictual troglobites, however, most species are poorly studied and others remain undiscovered. For example, Rambla and Juberthie [4] recognized a total of 82 troglobitic harvestmen worldwide, but that number is outdated and grossly underestimated. Harvestmen are common inhabitants of subterranean ecosystems and are well represented in all classification categories of subterranean fauna (sensu Trajano and Carvalho [5]). Troglobite harvestmen species could play an important role in understanding the complete phylogenetic history of their respective lineages. For example, the highly troglomorphic species *Jarmilana pecki* (Goodnight & Goodnight, 1977) from Belize was originally described in the Neotropical family Stygnommatidae, but new evidence, based on studies of genitalic morphology and molecular phylogenetic analyses, revealed that it was the first American representative of the Pyramidopidae, which was previously thought to be endemic to Africa [6]. The case of *Jarmilana pecki* clearly illustrates the challenges of interpreting systematic relationships when faced with highly convergent morphologies, and it emphasizes the need for an integrative approach (*e*.*g*., fine morphology, molecular phylogeny) to investigate the evolutionary history of troglobitic species.

Until the present study, the Brazilian troglobitic opiliofauna consisted of nine described species, all Laniatores–eight species of Gonyleptidae (*Discocyrtus pedrosoi* Kury, 2008; *Eusarcus elinae* Kury, 2008; *Giupponia chagasi* Pérez-González and Kury, 2002; *Iandumoema setimapocu* Hara and Pinto-da-Rocha, 2008; *Iandumoema smeagol* Pinto-da-Rocha, Fonseca-Ferreira and Bichuette, 2015; *Iandumoema uai* Pinto-da-Rocha, 1997; *Pachylospeleus strinatii* Šilhavý, 1974; *Spinopilar moria* Kury and Pérez-González, 2008) and one species of Escadabiidae (*Spaeleoleptes spaeleus* H. Soares, 1966). A number of other troglobitic harvestmen species are known, but are awaiting formal taxonomic description [7–9]. One species of Cyphophthalmi, *Canga renatae* DaSilva, Pinto-da-Rocha and Giribet, 2010, was collected exclusively from caves in the Brazilian state of Pará, but due to the absence of troglomorphic features, it was not considered a troglobite. The occurrence of this species in caves was attributed to the need for shelter from the dry climatic conditions in this region and it is assumed that the species also inhabits adjacent forests [10]. No troglobitic Eupnoi from Brazilian caves are known to date.

During surveys and studies of the Brazilian cave fauna, conducted by the "Laboratório de Estudos Subterrâneos" (Laboratory of Subterranean Studies) of the Federal University of São Carlos, Brazil, a tiny depigmented harvestmen species was collected from the Olhos d’ Água cave, a biospeleologically iconic Brazilian cave in Peruaçu, Itacarambi, Minas Gerais State. The specimens belong to a new genus and a new species of the family Kimulidae, herein formally described. A molecular phylogenetic analysis was used to test taxonomic hypotheses and provide a basis for a discussion of the biogeographic history of Kimulidae.

## Materials and methods

### Sampling locality

**Fig 1:**
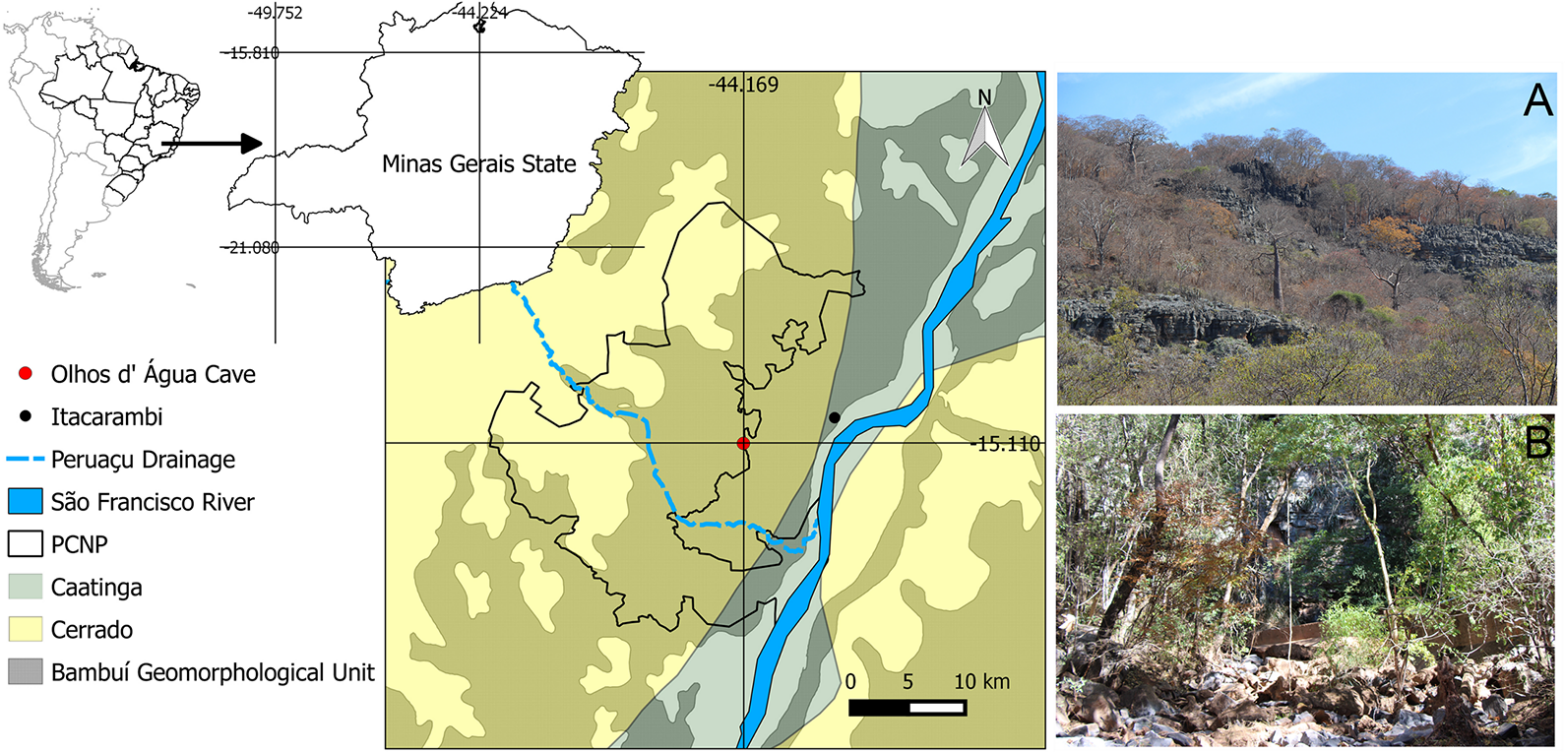
Locality of the Olhos d’Água cave in South America. (A), (B) Photographs of surrounding vegetation. PCNP: Peruaçu Caves National Park.

Olhos d’Água cave [geographic coordinates 15° 6'49.32"S, 44°10'10.56"W] is located in Peruaçu Caves National Park (PCNP), northern Minas Gerais State, Itacarambi municipality (Fig 1). The karst region of PCNP is composed of outcrops with a predominance of carbonate karst areas developed on Proterozoic rocks of the Bambuí Group, mainly limestone and dolomites (Januária/Itacarambi Formation sensu Piló and Kohler [11]).

Within the boundaries of the PCNP, the Peruaçu river runs through a valley with large walls, pipes and sinkholes forming its canyon. Peruaçu River Basin is a left tributary of São Francisco River (Piló and Kohler 1991). The PCNP is situated in the transition between the Cerrado and Caatinga morphoclimatic domains [12] and, according to the Koppen-Geiger classification [13], the climate is tropical semiarid, with a well-defined dry period between April and September, an average annual temperature of 24°C and average annual rainfall of 800 mm [14].

**Fig 2:**
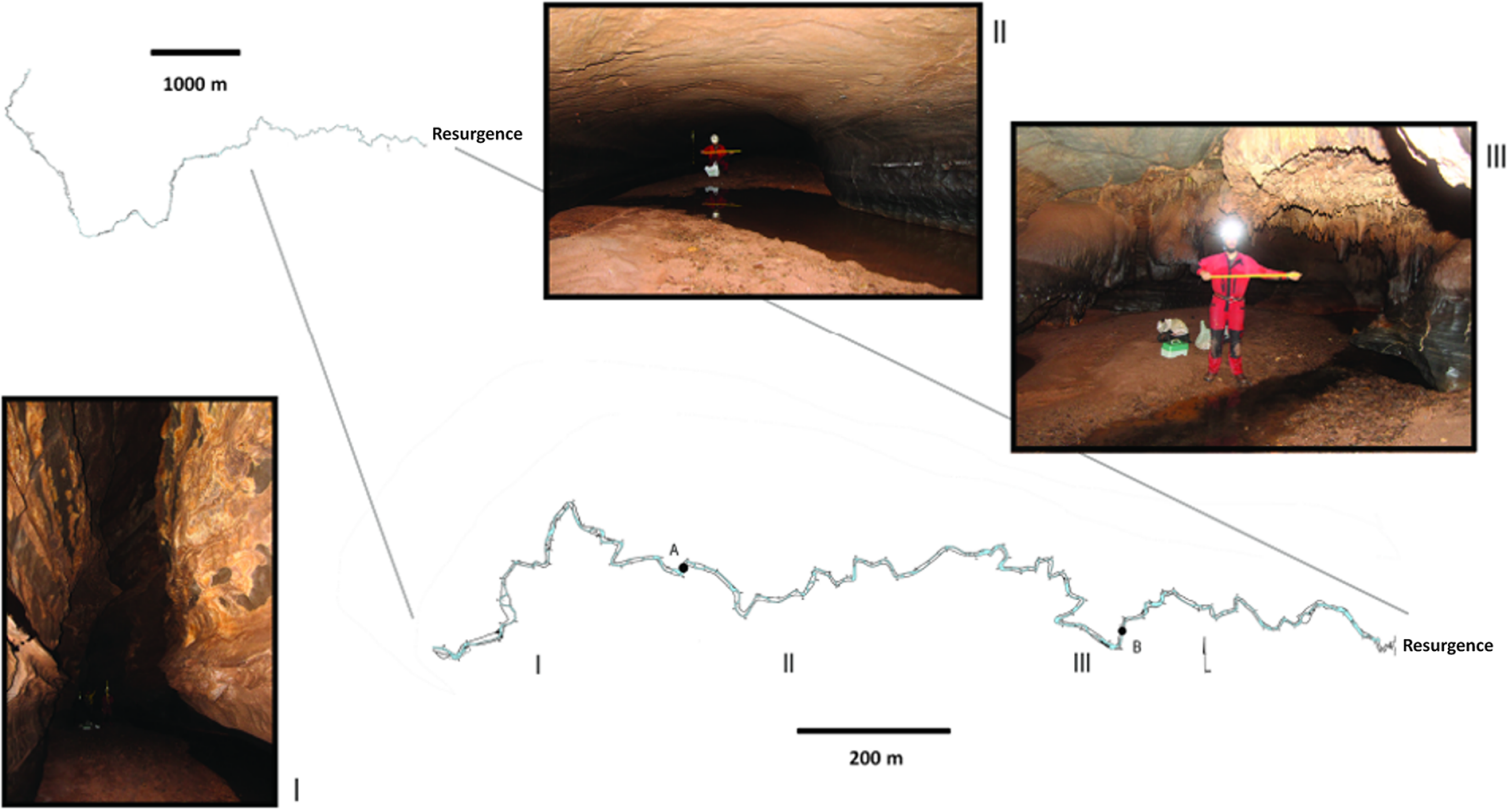
Cartographic map* of the Olhos d’Água cave. (I), (II), (III) Photographs corresponding to cave cross sections indicated on the detailed cartographic map. (A), (B) Sites in the cave where *Relictopiolus galadriel* gen. nov., sp. nov. was captured. * Courtesy of the Bambuí Speleological Group.

Olhos d’Água cave has a horizontal projection of approximately 9,100 m and consists of a long and sinuous conduit. The dimensions of galleries vary from low passages to large rooms, with few upper conduits. Although the area has the lowest amount of annual rainfall in the region, the drainage in the cave is perennial. On the other hand, intense flooding may occur in the cave during the rainy season. The main cave entrance is a resurgence, which is not located within the PCNP limits. The substrate and available microhabitats for the terrestrial fauna are predominantly formed by sand, boulders and other rocky substrates, silt, and with occasional vegetable debris deposited along the river banks (Fig 2).

### Specimen collection and repositories

The field study and collection of specimens at the Peruaçu Caves National Park (PCNP) were carried out under the SISBIO permit number 28992–3 (to MEB) issued by the Instituto Chico Mendes de Conservação da Biodiversidade (ICMBio) of the Ministério do Meio Ambiente (MMA), Brazil. The field study did not involve officially endangered or protected species. Specimens were collected by active search and were preserved in 80% and 96% ethanol for morphological study and DNA extraction, respectively. Types are deposited in the Laboratório de Estudos Subterrâneos, Universidade Federal de São Carlos, São Carlos, Brazil (LES/UFSCAR), Museu de Zoologia, Universidade de São Paulo, São Paulo, Brazil (MZUSP) and División de Aracnología in the Museo Argentino de Ciencias Naturales "Bernardino Rivadavia", Buenos Aires, Argentina (MACN-Ar). Other specimens used in this study are deposited at: Coleção de História Natura da Universidade Federal do Piauí (CHNUFPI), Universidade Federal da Paraíba (UFPB), Museu Nacional/Universidade Federal do Rio de Janeiro (MNRJ), Instituto Butantan, São Paulo (IBSP), National Museum of Natural History, Smithsonian Institution (NMNH), Zoologisk Museum Universität København (ZMUC), Naturmuseum Senckenberg Sektion Arachnologie (SMF).

### Maps

All maps were generated using the free, open source geographic information system software QGIS 2.16.3 (http://www.qgis.org/); the shape files are freely available from the Ministério do Meio Ambiente (MMA) web page (http://www.mma.gov.br/governanca-ambiental/geoprocessamento).

### Nomenclatural acts

This article conforms to the requirements of the amended International Code of Zoological Nomenclature. All nomenclatural acts contained within this published work have been registered in ZooBank. The ZooBank LSIDs (Life Science Identifiers) can be resolved and the associated information viewed by appending the LSID to the prefix “http://zoobank.org/”. The LSID for this publication is: urn:lsid:zoobank.org:pub:2ACD230C-33E9-4461-A40D-7FE418F94B21.

### Specimen preparations

Ethanol preserved specimens were photographed with a Leica DFC 290 digital camera attached to a Leica M165C stereomicroscope (at MACN), and different focal planes were combined using Helicon Focus Pro (www.heliconsoft.com). The picture of the holotype of *Tegipiolus pachypus* (SMF 9906896) was taken with a Sony DSC-V1 digital camera and no focal planes were combined. Color descriptions follow Kury and Orrico [15]. Male genitalia preparation follows Acosta et al. [16], with temporary mounts embedded in glycerol. Line drawings of male genitalia were made using a camera lucida attached to an Olympus BH-2 compound microscope (at MACN) and were digitized using Corel Draw X7. Figures were edited using Photoshop CS5 or Corel Draw X7. For SEM, dissected body parts were dried using Critical Point Drying and mounted on adhesive copper tape (EMS 77802; Electron Microscopy Sciences) affixed to an aluminum stub. Uncoated SEM preparations were examined using a FEI Quanta 250 (at the UFSCar).

### Taxon sample

Using the dataset from Cruz-López et al. [6] (88 terminals), we added new sequence data for 24 terminal taxa representing Escadabiidae (10 new terminals), Kimulidae (6 new terminals), Samoidae (3 new terminals), and Zalmoxidae (5 new terminals). The dataset was particularly strengthened by the addition of Escadabiidae and Kimulidae representatives and included more terminals of these Zalmoxoidea than all the previous molecular phylogenetic analyses of Laniatores. Specimen data for these 24 taxa included the molecular phylogenetic analysis, including voucher numbers and locality data, are listed in Table 1. Information for the rest of the terminals can be found in Sharma and Giribet [17] and Cruz-López et al. [6].

**Table 1:** Specimens included in this study for which new sequence data were obtained, including voucher numbers and collection details.

Family	Genus	species	Repository	DNA voucher	Name on the tree	Collection locality details
Escadabiidae	Gen. ind.	sp. ind.	MACN-Ar	Es01	Escadabiidae_Es01	ARGENTINA, Misiones Province, Parque Provincial Urugua-í, 25°48'32.94"S, 54° 1'0.70" W, 377 masl, 11.iii.2015, A. Pérez González, Daniel N. Proud, Sara Ceccarelli and A. Martinez-Aquino.
Escadabiidae	Gen. ind.	sp. ind.	MACN-Ar	Es02	Escadabiidae_Es02	ARGENTINA, Misiones Province, Parque Provincial Urugua-í, 25°48'32.94"S, 54° 1'0.70" W, 377 masl, 11.iii.2015, A. Pérez González, Daniel N. Proud, Sara Ceccarelli and A. Martinez-Aquino.
Escadabiidae	Gen. ind.	sp. ind.	MACN-Ar	Es03	Escadabiidae_Es03	ARGENTINA, Misiones Province, San Ignacio, Reserva Privada Osununú, January 2015, C.I. Paris, P.E. Hanish and A.F. Sanchez Restrepo.
Escadabiidae	*Brotasus*	*cf*. *megalobunus*	MACN-Ar	Es04	Brotasus_cf_megalobunus_Es04	BOLIVIA: Santa Cruz: Prov. Guarayos: Concesión Forestal La Chonta; S 15° 42' 42", W 62° 46' 20" 330 m. 26–30 Oct. 2010. Col. C. Grismado, M. R. Vacaflores & M. Pérez.
Escadabiidae	*Brotasus*	sp. ind.	LES	Es17	Brotasus_sp_Es17	BRAZIL, Mato Grosso State, Distrito da Cerquinha, munícipio de Nobres, Duto do Quebó, 14°26'45.7"S, 56°01'15.9"W, 330 masl, 23.ix.2015, M.E. Bichuette, A. Chagas-Jr, D.M. von Schimonsky. Epígeo. (field code MT 397).
Escadabiidae	*Baculigerus*?	sp. ind.	CHNUFPI 1094	Es06	Baculigerus?_sp_Es06	BRAZIL, Piauí, Castelo do Piauí, Fazenda Bonito, Espanha-Chile-Brasil Rochas Ornamentais do Brasil LTDA, S 5° 13'33.4", W 41°41'46.8", 10.v.2013, L.S. Carvalho *et al*.
Escadabiidae	Gen. ind.	sp. ind.	UFPB—OP0035	Es07	Escadabiidae_Es07	BRAZIL, Alagoas State, Mata do Brejo, Usina Serra Grande, São José do Laje, 8°58'48.00"S, 35°51'0.00"W, 16.ii.2012. A.M. DeSouza et al.
Escadabiidae	*cf*. *Spaeleoleptes*	sp. ind.	LES	Es08	cf_Spaeloleptes_Es08	BRAZIL, Bahia State, Ourolândia, Gruta da Fazenda Caldeirão, (11°00'56"S, 40°39'58"W), 772 masl, 17.vi.2015, J. E. Gallão. (field code GR055).
Escadabiidae	*Escadabius*	*ventricalcaratus*	UFPB AL-12	Es11	Escadabius_ventricalcaratus_Es11	BRAZIL, Pernambuco State, Reserva Particular do Patrimônio Natural Frei Caneca, 8°40'48.00"S, 35°51'0.00" W, 5-7.viii.2011, M.B. DaSilva et al. Leg.
Escadabiidae	*Baculigerus*	*cf*. *littoris*	MNRJ 08841	Es14	Baculigerus_cf_littoris_Es14	BRAZIL, Bahia State, Coração de Maria [12°15'17.55"S 38°48'29.33"W, 300 masl], Oct.2015, A. Kury & O. Villarreal Manzanilla.
Kimulidae	*Tegipiolus*	*pachypus*	UFPB AL-14	Es10	Tegipiolus_pachypus_Es10	BRAZIL, Pernambuco State, Caruaru, Parque Natural Municipal Professor João Vasconcelos Sobrinho (Brejo dos Cavalos), 8°21'36.00"S, 36° 1'12.00"W, 830 masl, x.2001, A.M. Souza & A. Lira.
Kimulidae	*Relictopiolus* **gen. nov.**	*galadriel* **sp. nov.**	LES	Es18	Relictopiolus_galadriel_Es18	BRAZIL, Minas Gerais State, Itacarambi, Peruaçu Caves National Park, Olhos d’ Água cave, 15° 6'49.32"S, 44°10'10.56"W, 24.x.2015, Monte, B.G.O., Zepon, T.
Kimulidae	*Relictopiolus* **gen. nov.**	*galadriel* **sp. nov.**	LES	Es19	Relictopiolus_galadriel_Es19	BRAZIL, Minas Gerais State, Itacarambi, Peruaçu Caves National Park, Olhos d’ Água cave, 15° 6'49.32"S, 44°10'10.56"W, 24.x.2015, Monte, B.G.O., Zepon, T.
Kimulidae	*Kimula*	sp. ind.	NMNH	Es21	Kimula_sp_Es21	CUBA, Guantánamo Province, Baracoa, Alejandro de Humboldt National Park, El Yunque, 20°20'42.04"N, 74°33'59.11"W, 370 masl, 5.iv.2012, CarBio Team. (CarBio field code CU-15)
Kimulidae	*Kimula*	sp. ind.	NMNH	Es22	Kimula_sp_Es22	PUERTO RICO, Cambalache, Bosque Cambalache, 3.iii.2012, CarBio Team. (CarBio field code PR-200).
Kimulidae	Gen. ind.	sp. ind.	MACN-Ar	Es24	Kimulidae_Es24	PANAMA: Coclé, Parque Nacional General de División Omar Torrijos Herrera, El Cope, 1 hectare PANCODING inventory [N 08°40'5.1", W 80°35'33.3"], 760 masl, 4-9.vi.2008, M. Arnedo, L. Benavides, G. Hormiga, F. Labarque, M. Ramírez Col. (field code STC1N6R)
Zalmoxidae	Gen. ind.	sp. ind.	MNRJ 08843	Es13	Zalmoxidae_Es13	BRAZIL, Bahia State, Vila Bela da Santíssima Trindade, Fazenda Barraco Alto [14°56'18.64"S, 60° 0'33.84"W, 222 masl], 14.xi.2015, A. Chagas-Jr. & A. Kury.
Zalmoxidae	Gen. ind.	sp. ind.	MNRJ 08842	Es15	Zalmoxidae_Es15	BRAZIL, Mato Grosso State, Porto Estrela, Estação Ecológica da Serra das Araras [15°30'3.26"S, 57° 9'57.35"W, 200 masl], 16.xi.2015, A. Chagas-Jr. & A. Kury.
Zalmoxidae	Gen. ind.	sp. ind.	LES	Es16	Zalmoxidae_Es16	BRAZIL, Mato Grosso State, Distrito da Cerquinha, munícipio de Nobres, Toca do Quati, 14°27'11.2"S, 55°59'12.6"W, 392 masl, 22.ix.2015, M.E. Bichuette, A. Chagas-Jr, D.M. von Schimonsky. Zona de entrada/declive-folhiço (field code MT 143).
Zalmoxidae	Gen. ind.	sp. ind.	NMNH	Es23	Zalmoxidae_Es23	PUERTO RICO, Villalba, Toro Negro, 18°10'22.72"N, 66°29'30.47"W, 889 masl, 27-29.vii.2011, CarBio Team (CarBio field code PR 17 CR)
Zalmoxoidea	Family and Gen. ind.	sp. ind.	NMNH	Op019	Zalmoxoidea gen sp. Op019	PUERTO RICO, Rio Grande, El Yunque, El Verde, 18°19'18.08"N, 65°49'11.67"W, 323 masl, 16-19.vii.2011, CarBio Team (CarBio field code PR001)
Samoidae	*Hummelinckiolus*	sp. ind.	NMNH	Op020	Hummelinckiolus_sp_Op020	CUBA, Santiago de Cuba Province, Siboney, 19°57'38.88"N, 75°42'27.36"W, 30 masl, 1.iv.2012, CarBio Team (CarBio field code CU007)
Samoidae	*Hummelinckiolus*	sp. ind.	NMNH	Op7048	Hummelinckiolus_sp_Op7048	DOMINICAN REPUBLIC, La Vega Province, Los Tablones, 19°03'18.58"N, 70°53'19.18"W, 1304 masl, 28.vi.2012, CarBio Team (CarBio field code DR030)
Samoidae	*Hummelinckiolus*	sp. ind.	NMNH	Op7050	Hummelinckiolus_sp_Op7050	DOMINICAN REPUBLIC, La Vega Province, Valle Nuevo, 18°50'46.79"N, 70°44'26.30"W, 2982 masl, 26.vi.2012, CarBio Team (CarBio field code DR022)

### DNA sequencing and alignment

DNA was extracted using the Qiagen DNeasy Blood and Tissue Kit, digesting tissue from one or two legs at 56° C over-night with Proteinase K and following the manufacturer’s protocol. The DNA was then used to amplify and sequence four molecular markers, namely fragments belonging to the mitochondrial cytochrome c oxidase subunit I (COI) gene, and the nuclear histone H3 (H3), 18S rRNA (18S) and 28S rRNA (28S) genes. Polymerase Chain Reactions (PCR) were carried out to amplify the four gene regions, setting up a master mix containing 1.5μl x10 PCR Buffer (Thermo Scientific), 10 μmoles MgCl_2_, 0.25 μmoles of each dNTP, 0.4 μmoles of each primer, 0.1 μl Taq Polymerase (Thermo Scientific), 0.5 μl BSA, 1–2 μl genomic DNA and ddH_2_O to bring the final volume to 15 μl. The primers used for amplification included LCO1490-HCOoutout (COI), H3aF-H3aR (H3), 1F-5R (18S), and 28Srd4.8a-28Srd7b1 (28S) following Sharma and Giribet [17]. Thermal cycling included an initial denaturing step at 95° C for 3 minutes, followed by 15 cycles of 30 seconds at 95° C, 30 seconds at the annealing temperature (51° C for nuclear and 45° C for mitochondrial gene fragments) and 45 seconds at 72° C; an additional 20 cycles were run with the annealing temperature lowered by 3° C and a final extension step of 10 minutes at 72°C was executed. PCR products were purified using ExosAP (Thermo Scientific) following the manufacturer’s protocol and sent for sequencing to Macrogen Inc., Korea. The chromatograms of the sequences were edited in Sequencher v. 4.1.4 (GeneCodes corp.), where the protein-coding gene fragments COI and H3 were checked for stop-codons.

The edited sequences were combined with sequences from previous studies [17,18] for a total of 124 ingroup and outgroup taxa. Alignments were constructed in the MAFFT v. 7 [19] web service, using the “Auto” strategy and a gap opening penalty of 1.53. For the protein-coding genes, COI and H3, their translation to amino acids was checked in MEGA 7.0.14 [20] and the hypervariable regions of 28S which could not be aligned with confidence were removed for the phylogenetic analyses using the “stringent” settings of the Gblocks server online [21]. Newly-generated sequences were submitted to GenBank and their accession numbers can be found in Table 2.

**Table 2:** GenBank accession numbers for newly-generated sequences.

Taxon	DNA voucher	COI	H3	18S	28S
Escadabiidae: gen. ind. sp. ind.	Es01	MF687049	MF687066	MF687005	MF687026
Escadabiidae: gen. ind. sp. ind.	Es02	MF687050	——	MF687006	MF687027
Escadabiidae: gen. ind. sp. ind.	Es03	MF687051	——	——	MF687028
Escadabiidae: *Brotasus cf*. *megalobunus*	Es04	——	MF687067	MF687007	MF687029
Escadabiidae: Brotasus sp.	Es17	——	MF687068	MF687008	MF687030
Escadabiidae: *Baculigerus*? sp.	Es06	MF687052	MF687069	MF687009	MF687031
Escadabiidae: Gen. ind. sp. ind.	Es07	——	——	MF687010	——
Escadabiidae: *cf*. *Spaeleoleptes* sp.	Es08	MF687053	——	——	MF687032
Escadabiidae: *Escadabius ventricalcaratus*	Es11	——	MF687070	MF687011	MF687033
Escadabiidae: *Baculigerus cf*. *littoris*	Es14	——	MF687071	MF687012	MF687034
Kimulidae: *Tegipiolus pachypus*	Es10	MF687054	——	MF687013	MF687035
Kimulidae: *Relictopiolus galadriel* **gen. nov.** *et* **sp. nov.**	Es18	MF687055	——	MF687014	MF687036
Kimulidae: *Relictopiolus galadriel* **gen. nov.** *et* **sp. nov.**	Es19	MF687056	——	MF687015	MF687037
Kimulidae: *Kimula* sp.	Es21	MF687057	MF687072	MF687016	MF687038
Kimulidae: *Kimula* sp.	Es22	MF687058	MF687073	MF687017	MF687039
Kimulidae: gen. ind. sp. ind.	Es24	——	——	——	MF687040
Zalmoxidae: gen. ind. sp. ind.	Es13	MF687059	MF687074	MF687018	MF687041
Zalmoxidae: gen. ind. sp. ind.	Es15	MF687060	——	MF687019	MF687042
Zalmoxidae: gen. ind. sp. ind.	Es16	——	MF687075	MF687020	MF687043
Zalmoxidae: gen. ind. sp. ind.	Es23	MF687061	MF687076	MF687021	MF687044
Zalmoxoidea gen. sp.	Op019	MF687062	——	MF687022	MF687045
Samoidae *Hummelinckiolus* sp.	Op020	MF687063	——	MF687023	MF687046
Samoidae *Hummelinckiolus* sp.	Op7048	MF687064	——	MF687024	MF687047
Samoidae *Hummelinckiolus* sp.	Op7050	MF687065	——	MF687025	MF687048

### Phylogenetic analysis and divergence time estimation

Phylograms and chronograms for our focal taxa and selected outgroups were obtained through Bayesian inference (BI). As site-specific nucleotide saturation can have a negative influence on phylogenetic inference, the nucleotide composition homogeneity was tested for each data matrix, including matrices of individual codon positions for the protein coding gene fragments. The program TreePuzzle [22] was used to evaluate nucleotide compositional homogeneity using a chi-squared metric. This test indicated that the third codon positions of both COI and H3 were highly saturated and were therefore excluded. Furthermore, the nucleotide substitution model and partitioning strategy for the combined (concatenated) data matrices was evaluated in PartitionFinder v. 1.1.1. [23] (for details see Table 3).

**Table 3:** Partitioning strategy and nucleotide substitution models selected for each partition by PartitionFinder for Bayesian phylogenetic analyses.

Partition	Substitution model	Reference
COI codon position 1	GTR+I+G	[24]
COI codon position 2	GTR+I+G	[24]
H3 codon position 1	GTR+I+G	[24]
H3 codon position 2	K81	[25]
18S rDNA	TIMef+I+G	[26]
28S rDNA	TrNef+I+G	[27]

A phylogram of the concatenated, partitioned data was obtained using Markov Chain Monte Carlo (MCMC) simulations in MrBayes v. 3.2.6 [28], with the corresponding substitution model set for each partition and unlinking all parameters. Two independent MCMC runs with four chains each were executed for 50 million generation, sampling every 5000 generations and, after checking for correct mixing of the chains and large enough effective sample sizes (>200), a consensus tree was built using a 10% burn-in, and nodal support was assessed through posterior probabilities (PP).

The time-calibrated tree was built in BEAST v. 1.8.3 [29] using the same partitioning strategy and nucleotide substitution models as in MrBayes. A birth-death prior was set for the trees, linked across the partitions to obtain a single topology. Clock priors were unlinked per marker and set as uncorrelated relaxed clocks with lognormal distributions and estimated rates. Node age estimates were based on a combination of fossil evidence and secondary calibrations. Three calibrations based on the estimated ages of fossils belonging to specific groups were set as minimum node age constraints. The first calibration was based on an available fossil, *Hummelinckiolus silhavyi* Cokendolpher and Poinar, 1998 [30], setting a minimum age of 16 Million years (Ma) for the most recent common ancestor (mrca) of the three *Hummelinckiolus* species in our dataset. A minimum age of 16 Ma was also set for the mrca of the three *Kimula* species in this study, based on the fossil *Kimula*? sp. [31]. Finally, based on a fossil of *Petrobunoides sharmai* Selden et al., 2016 [32], belonging to the family Epedanidae, a minimum age of 99 Ma was set for the mrca of the infraorder Grassatores minus the superfamily Phalangodoidea, given the sparse sampling of epedanids and the lack of resolution between said family and its sister groups in our phylogeny (see Results). The secondary calibration involved setting the minimum age of the zalmoxid taxa in this study to 87.3 Ma, based on the minimum range for the 95% highest posterior density (HPD) of the age obtained for this family by Sharma and Giribet [18]. With these priors, two independent MCMC runs were executed for 100 million generations each, sampling every 10,000 generations and combining the sampled trees from the two runs using LogCombiner (part of the BEAST package). After verifying that the ESS of all parameters was >200 in Tracer v. 1.5 [33], TreeAnnotator (part of the BEAST package) was used to choose the maximum clade credibility tree, with node information (posterior probabilities, 95% HPD of node ages etc.) based on all sampled trees minus 10% burn-in.

Phylogenetic analyses both in MrBayes and BEAST were repeated including the third codon positions of COI and H3 to corroborate the effect of their initial exclusion on topology, nodal support and node age estimates.

## Results and discussion

### Phylogeny

The information for the DNA sequence data matrices used for the phylogenetic inferences in this study can be found in Table 4.

**Table 4:** Nucleotide composition of the four DNA data matrices of the markers used for phylogenetic inferences in this study, showing the number of taxa (ntax), the total number of sites in the alignment (nsites) and the number of variable (nvar) and parsimony-informative (npis) sites.

Marker	ntax	Nsites	Nvar	npis
COI	85	816	548	494
H3	87	327	142	136
18S	122	1769	257	257
28S	123	2174	498	325

**Fig 3:**
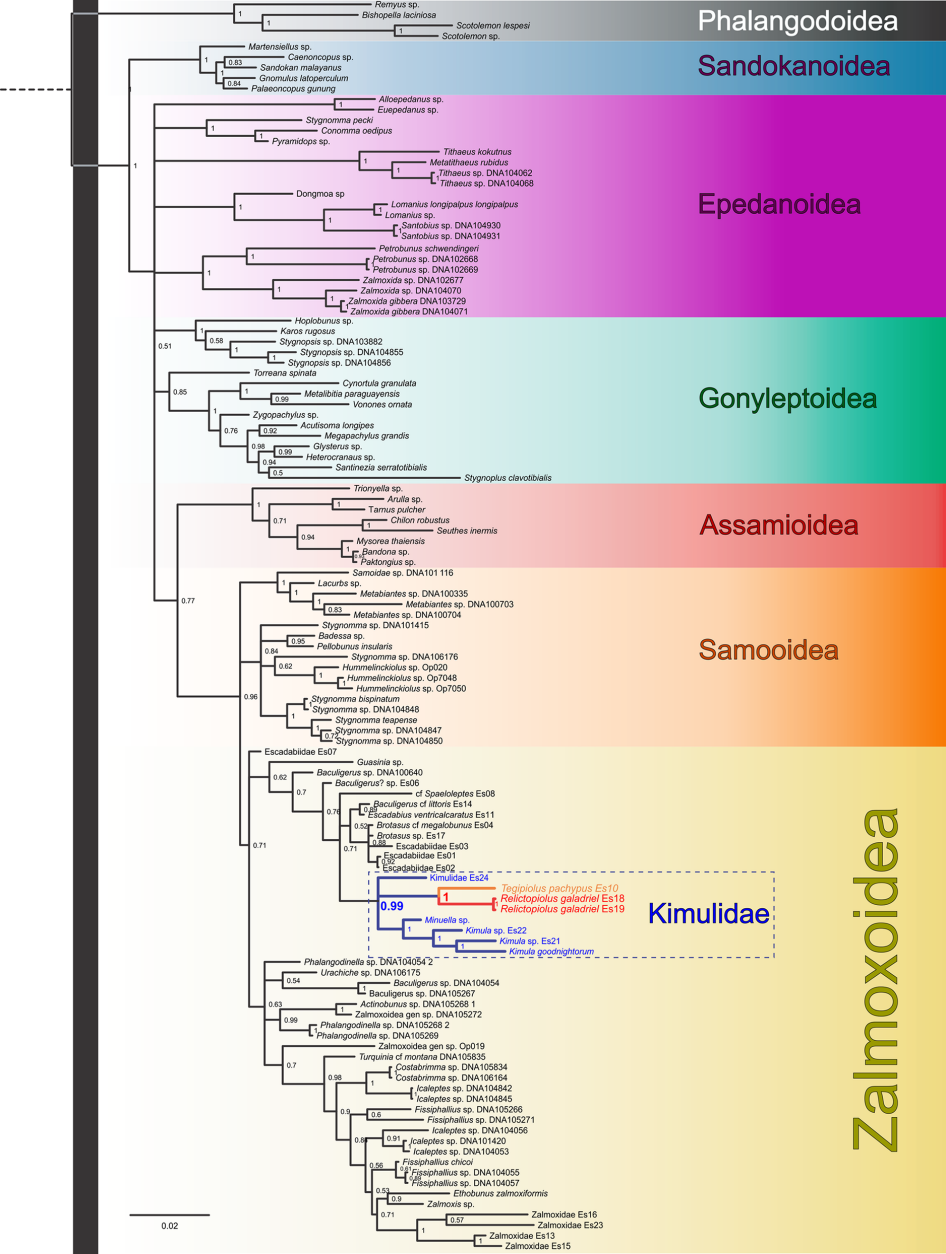
Phylogram of Grassatores obtained by Bayesian inference analysis, conducted in MrBayes, using the complete concatenated dataset. Support values at nodes represent posterior probabilities (PP). Nodes with PP<0.50 are collapsed. Colors represent the current superfamilial divisions (sensu Fernandez et al. [35]). The monophyletic family Kimulidae is enclosed in a box.

**Fig 4:**
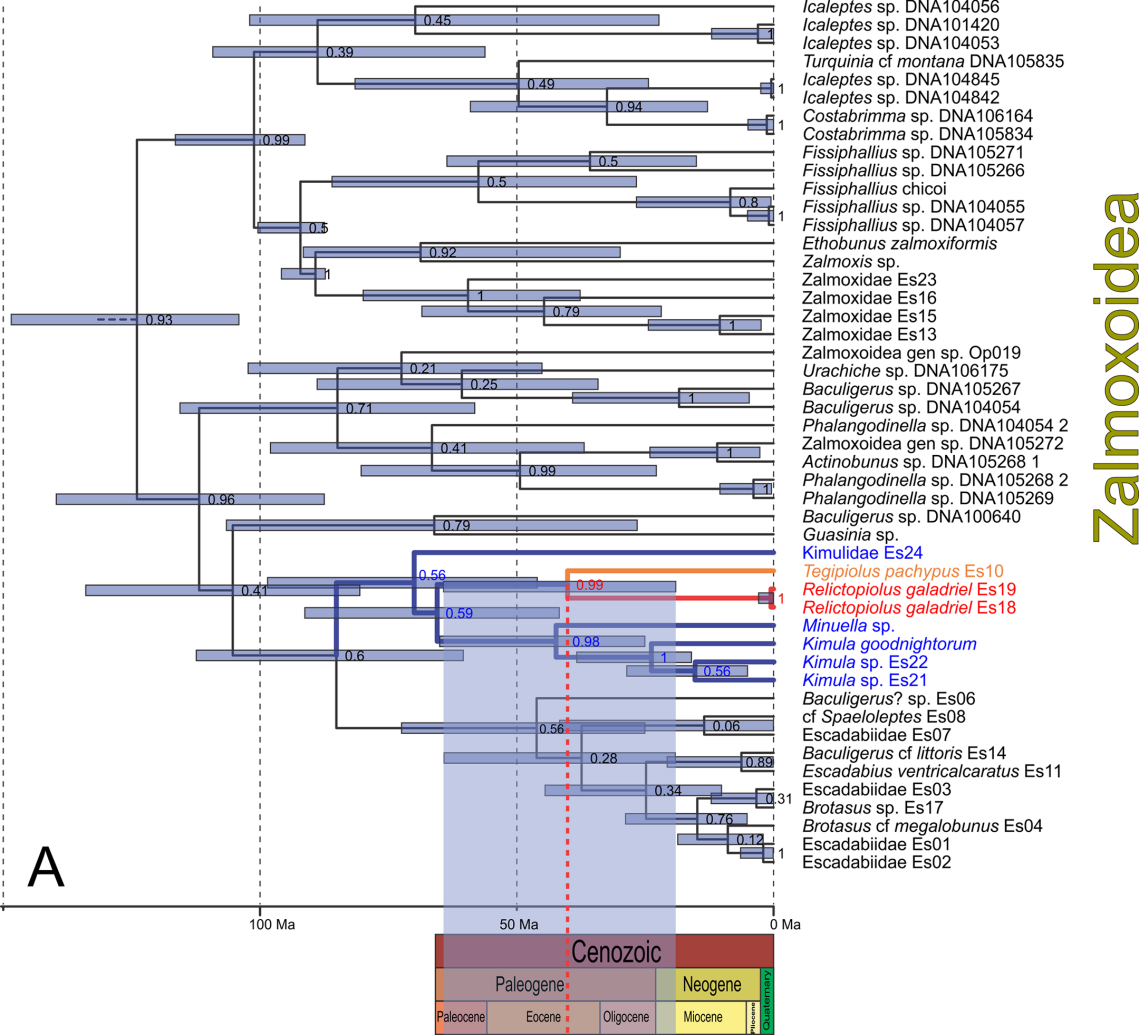
Chronogram (*partim*, Zalmoxoidea) obtained by Bayesian inference analysis, conducted in BEAST, using the complete concatenated dataset. (A) Chronogram with support values at nodes represent posterior probabilities and blue bars represent the 95% Highest Posterior Densities (HPD) around the estimated age of the most recent common ancestor. Colored branches and names of taxa represent the family Kimulidae. Below the chronogram, a geologic time scale of the Cenozoic Era (present-65 Ma) is shown. The vertical red-dotted line indicates the divergence time (40.1 Ma) between *Relictopiolus galadriel* **gen. nov.**, **sp. nov.** and *Tegipiolus pachypus* Roewer, 1949, projected over the geologic time scale with a blue shaded box that indicates the 95% HPD.

The phylogenetic topologies obtained by Bayesian Inference implemented in MrBayes (phylogram; Fig 3, S1 Fig) and BEAST (chronogram; Fig 4, S2 Fig) were similar, although certain nodes differed in whether the monophyly of the group was supported by posterior probabilities, or not. Similarly, there were no substantial topological or node-age differences when the saturated (COI and H3 third codon positions) data was included (S3 and S4 Figs), except that with the latter datasets, the overall nodal support values were lower. We therefore restrict the presentation and discussion of results to the phylogenetic trees obtained with the datasets excluding third codon positions. Discussions of the higher-level systematic of Laniatores are beyond the scope of the present contribution, but some findings do warrant a brief mention. Our phylogram (Fig 3) indicates strong support for the monophyly of all non-phalangodid Grassatores (PP = 1.0) corroborating previous findings that have utilized this dataset (*e*.*g*. [6,17,34]). One remarkable congruence between our phylogram and the Opiliones transcriptomic-based phylogenetic hypothesis of Fernández et al. [35] is the the sister group relation of Assamioidea with the Samooidea+Zalmoxoidea clade (albeit with low support, PP = 0.77). The chronogram failed to recover this relationship, placing Assamiidae as sister to Pyramidopidae, but with no support (PP = 0.2). Also consistent with previous studies [6,17,35,36], we recovered the clade Samooidea+Zalmoxoidea with high support (phylogram with PP = 0.96 and chronogram with PP = 1) despite the unstable internal relationships of this clade (Figs 3 and 4).

To achieve the objective of our study, and test our hypothesis regarding the familial placement of the new troglobitic species, we generated a dataset to include the broadest taxon sampling of the species diversity for Kimulidae and Escadabiidae to date, adding 16 new terminals. We obtained strong support for the monophyly of Kimulidae based on the MrBayes analysis but not for the BEAST analysis (PP_MRBAYES_ = 0.99; PP_BEAST_ = 0.56). Nested within the monophyletic Kimulidae was our focal taxon, the troglobite *Relictopiolus galadriel* **gen. nov.**, **sp. nov**., recovered as the sister-taxon to *Tegipiolus pachypus* Roewer, 1949, with strong support (PP_MRBAYES_ = 1.0; PP_BEAST_ = 0.99). The age estimate for the most recent common ancestor of these two species is 40.12 million years ago (Ma; 95% HPD: 19.06–64.27) (Fig 4) suggesting that they diverged sometime during the Paleogene.

Despite our efforts to greatly increase the taxon sampling within these zalmoxoid lineages, the sister-group of Kimulidae remains unclear, possibly obscured by the poorly understood diversity of the Escadabiidae which were not recovered as monophyletic in either analysis. However, the maximum clade credibility tree from BEAST places Kimulidae as sister to a monophyletic Escadabiidae, albeit without valid nodal support (Fig 4). Careful examination of morphological characters (e.g., genitalia) and a denser taxon sampling will be necessary to study the deeper phylogenetic relationships within these families, and their relationships within the Samooidea+Zalmoxoidea clade.

### Disjunct distributions

**Fig 5:**
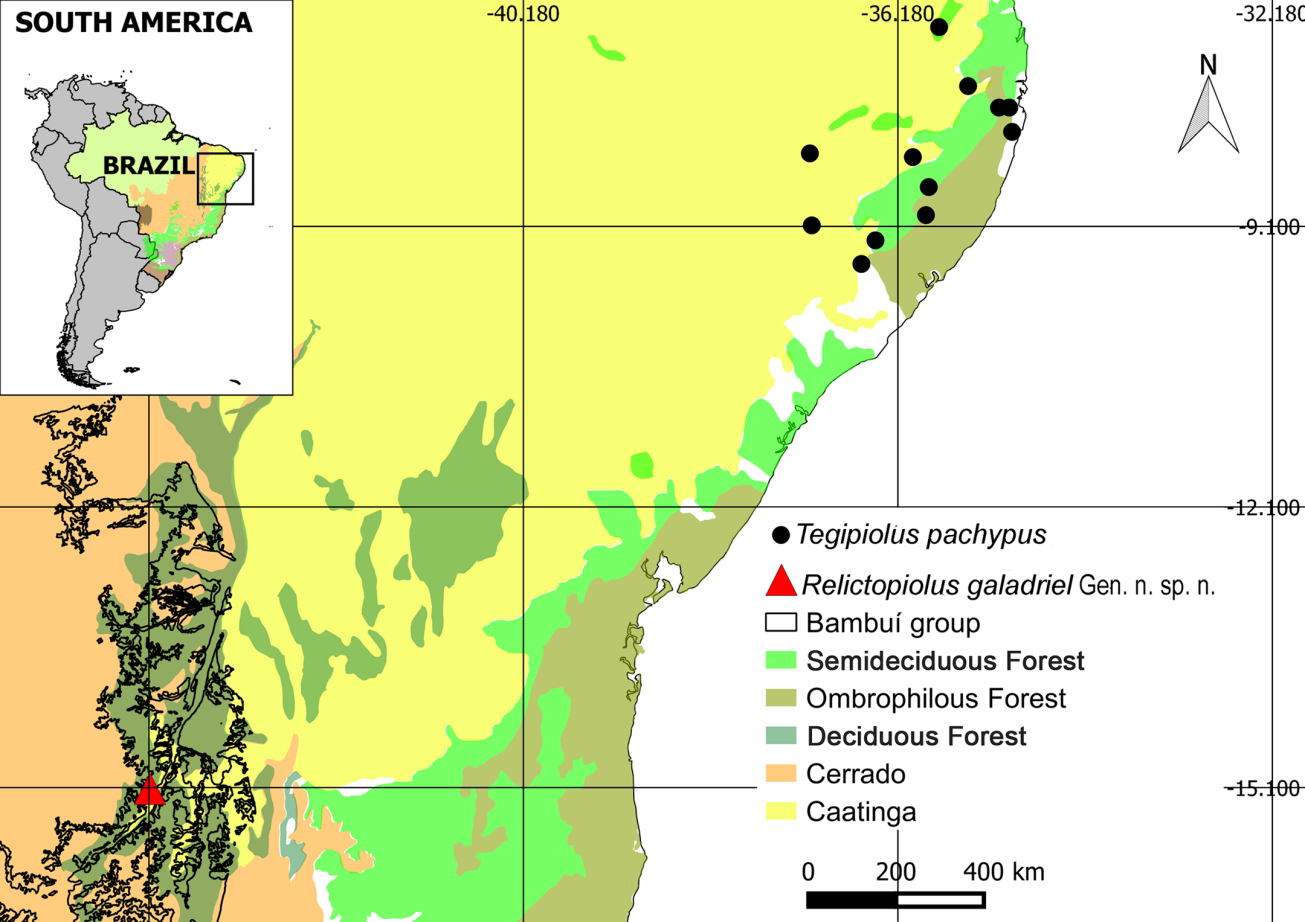
Current distribution of *Relictopiolus galadriel* gen. nov., sp. nov. (triangle) and *Tegipiolus pachypus* Roewer, 1949 (dots). The vegetation types (sensu the vegetational classification system proposed by the Instituto Brasileiro de Geografia e Estatística [37]) are delimited by color.

The Olhos d’Água cave, the type-locality of *Relictopiolus galadriel* **gen. nov.**, **sp. nov.**, is situated along the transition zone between the Caatinga and Cerrado ecoregions and is surrounded by a shrubby and dry vegetation (Figs 1A, 1B and 5). However, the vegetation classification system indicates a deciduous forest [37] covering the limestone lithology of the region and following the São Francisco river, which is considered part of the Atlantic Forest biome. Historical biogeography of biota living in this region indicates a relationship between the area of endemism of the eastern Atlantic forest of Bahia [38,39] and the south-eastern Amazon forest, as these two large blocks of Neotropical rain forests were once connected [38].

Approximately 1000 km to the northeast of the Olhos d’Água cave, in the northeastern regions of the Brazilian Atlantic Rain Forest biome, *T*. *pachypus* inhabits coastal humid forests and inland semidecidous forests (Fig 5). The coastal plain and the eastern slopes of the plateau (Serra da Borborema) are covered by Ombrophilous Forest, characterized by a wet climate, while the interior and northern regions have semideciduous forests that experience a marked dry season. Records indicate that the species can be found in semideciduous enclaves of mesic forest at higher altitudes (> 600 m), known as "Brejos de Altitude", but not in the surrounding lowland habitats of dry Caatinga scrubland. The range of *T*. *pachypus* coincides with the ranges of many animals and plants that are endemic to the Pernambuco interior and coastal forests [40] and thus appears to be related to the Bahia area of endemism [41,42].

**Fig 6:**
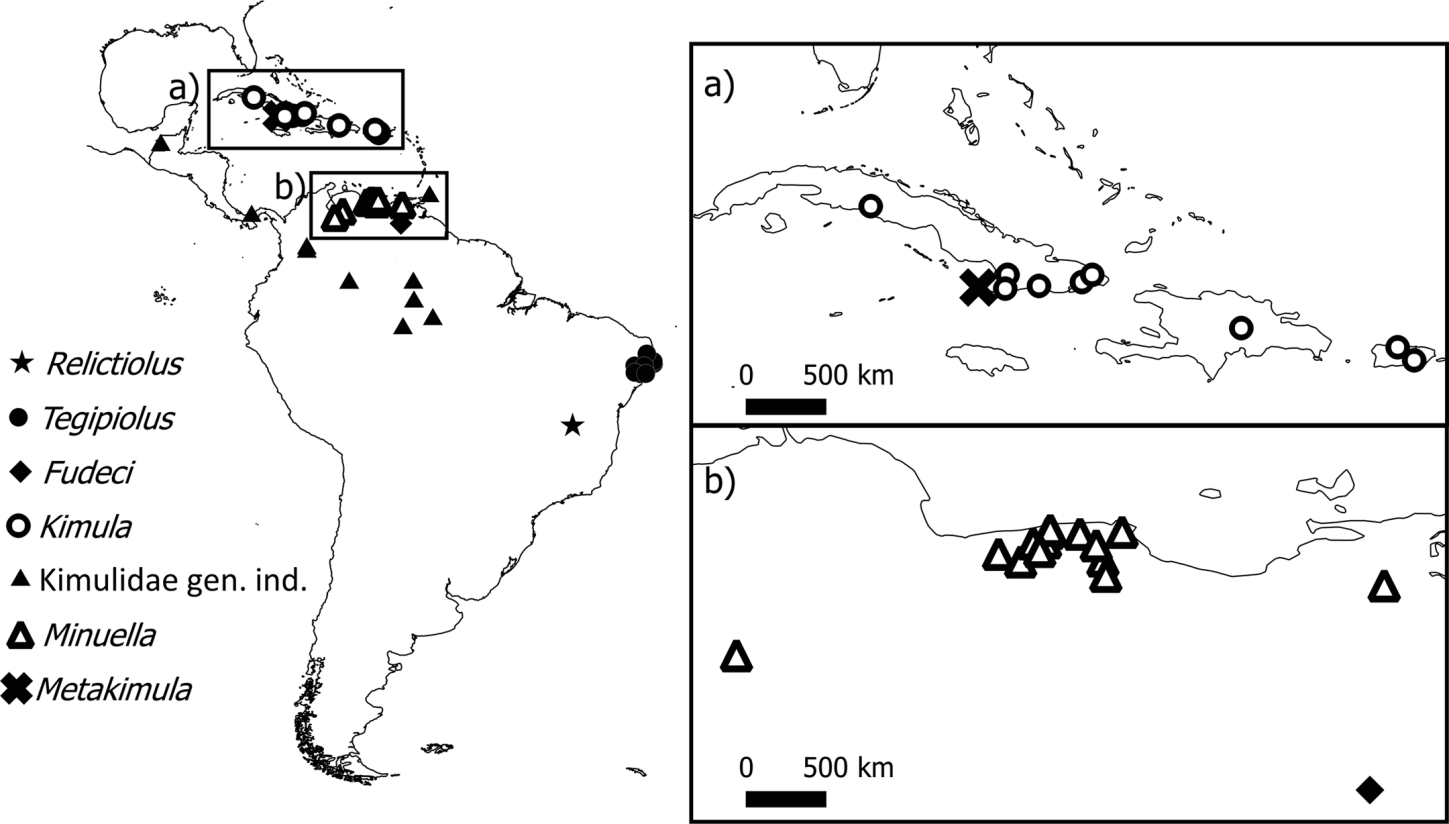
Known distribution of the family Kimulidae detailing the geographic range of each genus.

While the 1000 km expanse that separates *Relictopiolus* and *Tegipiolus* (Fig 5) is quite astonishing, what is more remarkable is the geographic disjunction between these sister-taxa and all other Kimulidae, which exhibit peak diversity in northwestern South America and the West Indies (Fig 6). Specifically, the kimulid genera *Minuella* Roewer, 1949 and *Fudeci* González-Sponga, 1998, occur in Venezuela, mainly in humid and high-altitude habitats, and *Kimula* Goodnight and Goodnight, 1942 and *Metakimula* Avram, 1973, occur in Antillean islands (Cuba, Puerto Rico, Hispaniola and Virgin Islands). A representative of Kimulidae (undetermined genus and species) is herein recorded for Panama and other unpublished records denote the presence of this family in Chiapas, Mexico (representing the northern limit of the family in the continental Americas), Trinidad and Tobago (MCZ Collections Database, http://mczbase.mcz.harvard.edu/) Colombia and Brazil (Manaus and Coari—360 Km W of Manaus—Amazonas State, Pío Colmenares pers. comm; Caracaraí, Roraima State, MCZ Collections Database, http://mczbase.mcz.harvard.edu/) (Fig 6). Thus, geographically, the nearest kimulid species to *Relictopiolus* and *Tegipiolus* is located more than 2,000 km to the northwest in Manaus, Brazil.

### Biogeography of Kimulidae

The phylogenetic relationships of Kimulidae appear to reflect the general biogeographic hypotheses of the major Neotropical areas defined by Morrone [43]. We recovered a clade consisting of *Kimula* and *Metakimula* from the Antilles and *Minuella* from Venezuela, illustrating a shared evolutionary history between the Antillean subregion and Pacific dominion of sensu Morrone [44]. An undetermined genus from Panamá, which undoubtedly belongs to Kimulidae, was not recovered as the sister group to the Venezuelan taxa (Fig 4). This suggests that there is a complex evolutionary history for Kimulidae in the Andean region, but broader taxon sampling is required. The distributional ranges of *Relictopiolus* and *Tegipiolus* fall within the Paraná dominion of Morrone’s classification [44], which is composed of Atlantic Forest biota. Fauna in the Atlantic Forest is related to that of the South-eastern Amazonia and Chacoan dominions, represented by the drier Caatinga and Cerrado biomes [44,45]. Thus, the two major clades of Kimulidae inhabit biogeographic regions that are not directly related in the context of evolutionary and biogeographic history in South America [43].

There are at least two plausible, nonexclusive explanations for the biogeographic patterns observed for *Relictopiolus* and *Tegipiolus*: 1) that we have a poor understanding of the diversity of Kimulidae and its species distributions, and 2) that these two species represent relicts of a once widespread ancestral distribution that has undergone range reduction due to extinction. First, there is undoubtedly a large gap in our taxonomic knowledge of this group. There may be myriad species inhabiting tropical forests in the southern and eastern parts of the Amazon Rainforest that remain undiscovered and undescribed. However, in the Amazon Forest near Manaus, after conducting multi-year intensive surveys within a large reserve, Kimulidae appear to be extremely rare—only a single species was discovered, based on a single specimen (Pío Colmenares pers. comm.). Additionally, of the 71 species recorded for the Brazilian state of Amazonas and 45 species recorded for the state of Pará, there are no known species of Kimulidae [46]. Therefore, although the list of known species is by no means exhaustive in these two states, the paucity of records for even a single species for Kimulidae leads us to hypothesize that these two taxa represent a case of relictualism.

### Biogeography of *Relictopiolus + Tegipiolus*

The split between *Relictopiolus* and *Tegipiolus* also appears to result from relictualism. The 1000 km expanse that separates these species is occupied by the Caatinga bioregion, a semiarid barrier (Fig 5). Based on natural history observations and available records, species of Kimulidae appear to primarily occupy and thrive in moist environments. Thus, the occurrence of *Relictopiolus* may be a relict of a widespread ancestral distribution throughout tropical forests that stretched from the Amazon to the Bahia Atlantic Forest–covering what are now the semiarid habitats of the Caatinga with tropical habitats. As with most harvestmen taxa that exhibit peak diversity in tropical regions, the Kimulidae are poorly studied and the diversity of the family is extremely underestimated. There are many species that remain to be described, and only then can we begin to formulate and test stronger hypotheses regarding the systematics and biogeography of this family and its interfamilial relationships within Zalmoxoidea.

The geographic distance between *Tegipiolus* and *Relictopiolus* is correlated with a deep genetic divergence that is estimated to have occurred during the Paleogene, 40.1 Mya (95% HPD: 19.1–64.3 Mya) (Fig 4, Paleocene to early Miocene). Since the origin of angiosperm plants during the Triassic, the most favorable climatic conditions for evergreen tropical vegetation in South America seems to have been during the early to middle Eocene [47]. There were two particularly noteworthy intense warming events in the Cenozoic: the short, Paleocene-Eocene Thermal Maximum (PETM) beginning at 55.8 Ma and lasting for approximately 200-ka, and the longer, Early Eocene Climatic Optimum (EECO) lasting 2–4-Ma, when tropical temperatures reached ∼32–34°C [48–50]. The peak of the EECO occurred from 53 to 50 Ma as the culmination of a prolonged period of global warming and climatic change [51]. It was accompanied by substantial shifts in greenhouse gas concentrations, global temperatures, and precipitation patterns, as well as floral and faunal biogeographies. Reconstructions of terrestrial climatic and environmental conditions, based on data from the Southern Hemisphere, suggest that the EECO was marked by peak period of carbon isotope enrichment (up to 5% higher), increased mean annual temperature (up to 6°C higher), and increased mean annual precipitation (up to 500 mm yr^–1^ higher) [52]. The increase in temperature has been associated with a significant increase in tropical plant diversity (∼30%) [48,53,54] correlated with an Eocene radiation of terrestrial biota including arthropods such as leaf-cutter ants [55] evidenced a rich tropical rain forest in South America at that time. In Brazil, a Tropical Rainforest seems to have been the dominant biome during the Paleocene/Eocene according to fossil records from Brazilian deposits in Ipixuna/Para State, Maria Farinha/Pernambuco State and Bacia Itaboraí/Rio de Janeiro State [56] and the Olhos D’Água cave was likely to be surrounded by tropical rainforests during those periods. Thus, we can hypothesize that the range of Kimulidae was much more widespread in eastern Brazil during the Eocene, specifically the most recent common ancestor of the *Tegipiolus* + *Relictopiolus* clade. During the Oligocene to Miocene, cooler and drier climates caused a retraction of rainforest biomes while savannahs (e.g., Cerrado) and other less humid biomes flourished in South America [57]. This would have resulted in the constriction of ancestral ranges of biota that were restricted to warm, humid habitats. Thus, it appears that *Relictopiolus galadriel* is a product of relictualism best explained by an Eocene radiation of Kimulidae in South America in which a single troglobitic lineage inhabiting the climatically stable, aphotic habitat inside the Olhos d’Água cave survived while the closest epigean relatives were driven to extinction by the changing climatic and environmental conditions surrounding the cave. The finding of this new Brazilian troglobite lends further support to the hypothesis that the Olhos d’Água cave acted as a stable refugium for myriad fauna, including this ancient lineage of Opiliones, and serves as an exemplary case of a hipogean habitat that represents a "museum" of biodiversity preservation (sensu Stebbins [58]) similar to the Australian Wet Tropics for Cyphophtalmi harvestmen [59] and Western and Central African forests for Ricinulei [60].

### Systematic account

**Order OPILIONES Sundevall,** **1833**

**Suborder LANIATORES Thorell,** **1876**

**Family KIMULIDAE Pérez-González, Kury and Alonso-Zarazaga *in* Pérez-González and Kury** **2007**

**Type genus.** *Kimula* Goodnight and Goodnight, 1942.

**Included genera.** *Fudeci* González-Sponga, 1998, *Kimula* Goodnight and Goodnight, 1942, *Metakimula* Avram, 1973, *Minuella* Roewer, 1949, *Tegipiolus* Roewer, 1949 and *Relictopiolus* Pérez-González, Monte and Bichuette, **gen. nov.** (See previous family composition in [61]).

**Excluded genera (herein transferred to Zalmoxidae).** *Acanthominua* Sørensen, 1932, *Euminua* Kury and Alonso-Zarazaga, 2011, *Euminuoides* Mello-Leitão, 1935 and *Pseudominua* Mello-Leitão, 1933.

*Genus* ***Relictopiolus** Pérez-González, Monte and Bichuette, **gen. nov.** urn:lsid:zoobank.org:act:AF21302A-6284-455E-AF0F-D989040E07D0*

**Type species.** *Relictopiolus galadriel* **sp. nov.**

**Included species.** monotypic.

**Etymology.** The genus name is a combination of "relicto"(from Latin *relictus*, past participle of *relinquere* 'leave behind'), referring to something that has survived from an earlier period or in a primitive form, and "*piolus*" indicating its relationship to *Tegipiolus*, the sister genus. Gender masculine.

**Comparative diagnosis.** Individuals of *Relictopiolus* **gen. nov.** are the smallest Kimulidae (1–1.1 mm body length) sharing the small size (less than 2 mm) with *Tegipiolus* Roewer, 1949 and *Fudeci* González-Sponga, 1998, whereas a group of ‘large-bodied kimulids’ consists of *Kimula* Goodnight and Goodnight, 1942, *Metakimula* Avram, 1973 and *Minuella* Roewer, 1949 (median 5.3 mm body length, ranging from 3.06–6.9 mm). Penis morphology of *Relictopiolus* **gen. nov.** resembles that of *Tegipiolus* but clearly differs from this and all other kimulid genera by the following characteristics: *Pars distalis* with the lamina ventralis partially surrounding the glans, latero-dorsally with three pairs of huge, wide and flattened macrosetae, conductors laminar expanded apically (hammer-like), wide rounded stylus surrounded by a thin ring-like parastylar collar. External morphology is similar to *Tegipiolus* and together they differ from other kimulids by the broad base of the ocular tubercle (ocularium); the thick and massive spiniform apophyses on the antero-lateral border of carapace; and the mesotergal scutum with vestigial/incomplete sulci, except a deep, well-marked sulcus I and a shallower sulcus V. Additionally, in males of *Relictopiolus* **gen. nov.** and *Tegipiolus*, all mesotergal areas are approximately the same width (or slightly wider at areas IV–V) whereas in the ‘large-bodied kimulids’ the widest part of the mesotergum is at the level of areas I–II. The other tiny kimulid, *Fudeci*, also has the wider mesotergal portion at the level of areas IV–V, but it clearly differs from *Relictopiolus*/*Tegipiolus* by the absence of an enlarged and armed femur IV, the absence of the strong spiniform apophyses on the antero-lateral border of carapace, the absence of armature on the free tergites and the morphology of the ocularium. *Relictopiolus* **gen. nov.** differs from *Tegipiolus* by the following external features: bell-shaped scutum magnum with the carapace wider relative to the mesotergum; posterior ocularium region of the carapace armed with two obtuse granulated setiferous tubercles; mesotergal areas with low, wide and blunt median granulated setiferous tubercles; femur IV less swollen; and troglomorphic features including anophthalmia and loss of pigmentation in the cuticle. Details of the exomorphological characteristics are provided in the species description below.

**Remark:** *Relictopiolus* **gen. nov.** and *Tegipiolus* are closely related, but have several important exomorphological differences. These differences do not single-handedly justify the erection of another monotypic genus, and based on these data *Relictopiolus* could be interpreted as a highly modified troglomorphic member of *Tegipiolus*. Thus, in addition to exomorphology, our decision to erect a new genus is supported by several remarkable differences in male genitalia including: i) glans partially surrounded by lamina ventralis, whereas in *Tegipiolus*, glans fully surrounded by lamina ventralis; ii) lamina ventralis contiguous instead of separated into two halves; iii) lamina ventralis laterally flat rather than strongly convex; iv) three pairs of macrosetae, rather than four; v) circumpenial concave fold absent in *Relictopiolus*, but strongly developed in *Tegipiolus* and located below the group of macrosetae; vi) conductors apically hammer-like instead of apically rounded; vii) two small pointed setae located ventrally, instead of laterally; viii) tubular stylus with a rounded tip instead of an enlarged tip. The gross differences in the penial groundplan between *Relictopiolus* and *Tegipiolus* are of the same magnitude as those which separate other kimulid genera such as *Kimula*, *Metakimula* and *Minuella*. Furthermore, the intraspecific variation observed in the external and genital morphology between geographically isolated populations of *Tegipiolus pachypus* may correspond to a species complex, but that is beyond the scope of the present contribution and should be addressed in a future work.

**Distribution.** Endemic to Peruaçu Caves National Park, Minas Gerais State, Brazil.

***Relictopiolus galadriel*** *Pérez-González*, *Monte and Bichuette*, ***sp*. *nov*.** *urn*:*lsid*:*zoobank*.*org*:*act*:*C115B21E-BE88-49A5-8813-DC77D36B8EF4*

(Figs 7–17).

**Fig 7:**
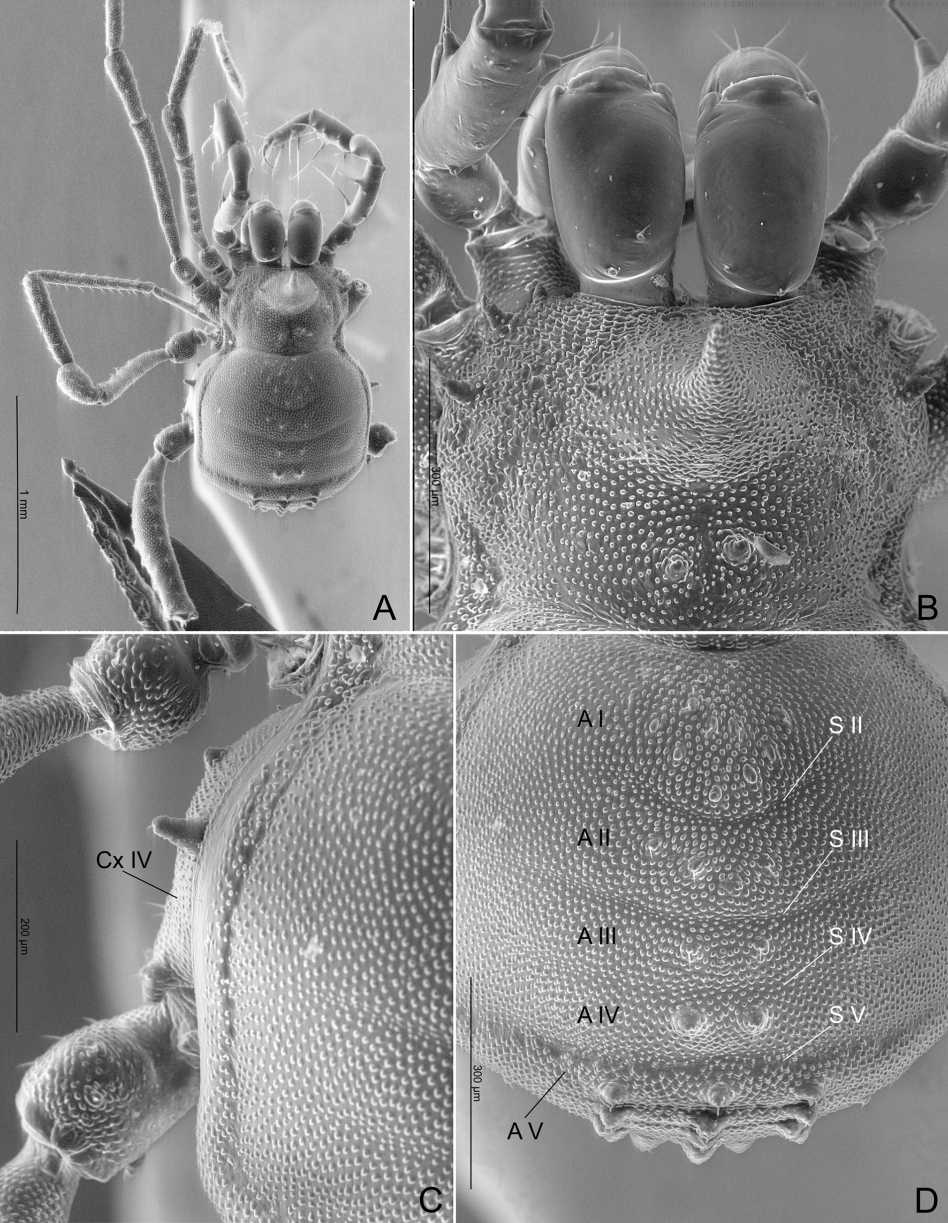
*Relictopiolus galadriel* gen. nov., sp. nov. male paratype (LES/UFSCar 0011189), dorsal view. (A) Habitus. (B) Detail of carapace and chelicerae. (C) Detail of left coxa IV. (D) Detail of mesotergal scute. A I‒V: areas I to V; Cx IV: coxa IV; S II‒V: sulci II to V.

**Fig 8:**
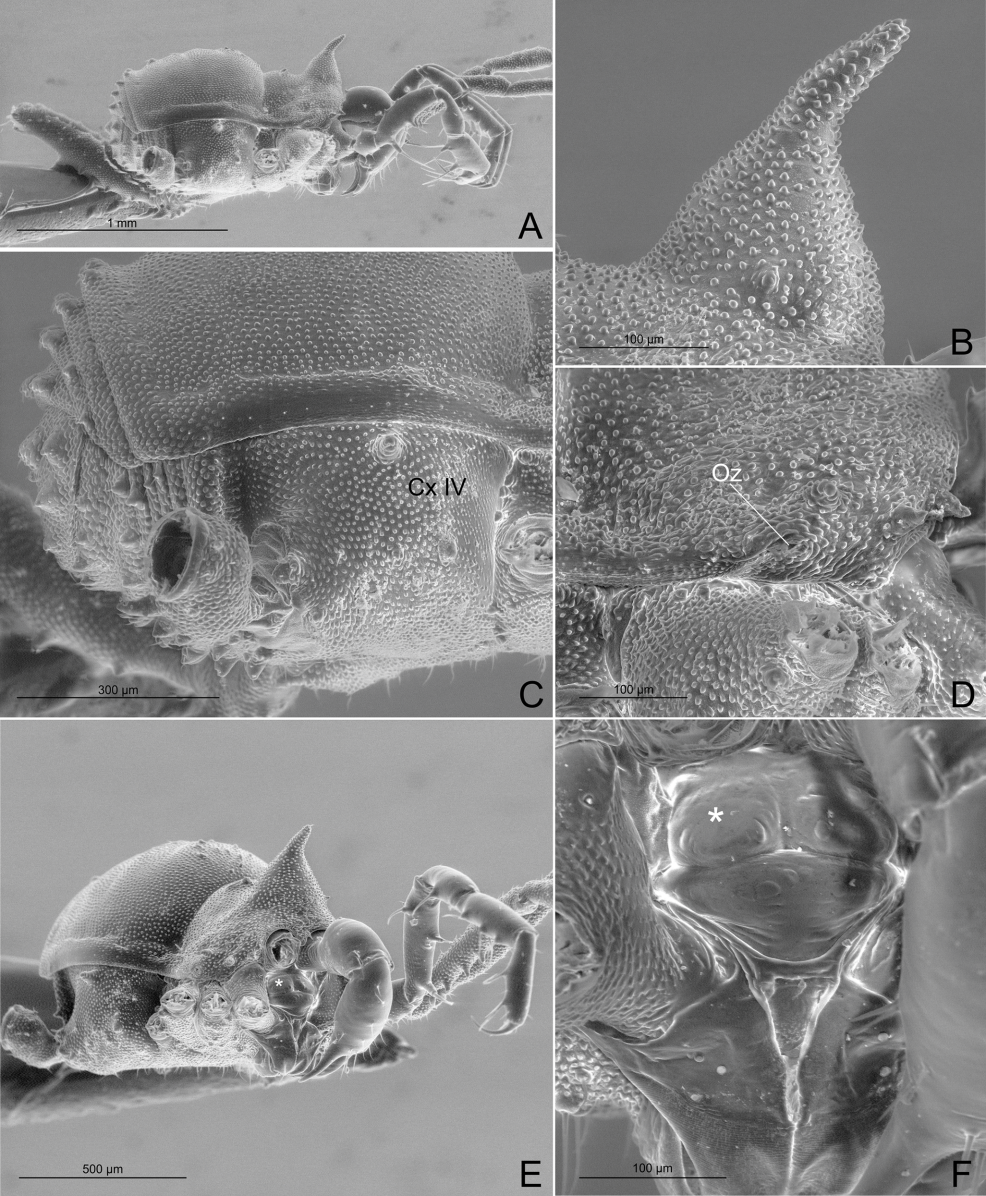
*Relictopiolus galadriel* gen. nov., sp. nov. male paratype (LES/UFSCar 0011189). (A) Habitus lateral view. (B) Detail of ocularium, lateral view. (C) Detail of right coxa IV, lateral view. (D) Antero-lateral carapace showing the ozopore region. (E) habitus latero-frontal view. (F) Detail of epistome, frontal view. Cx IV: coxa IV; Oz: ozopore; *: epistome.

**Fig 9:**
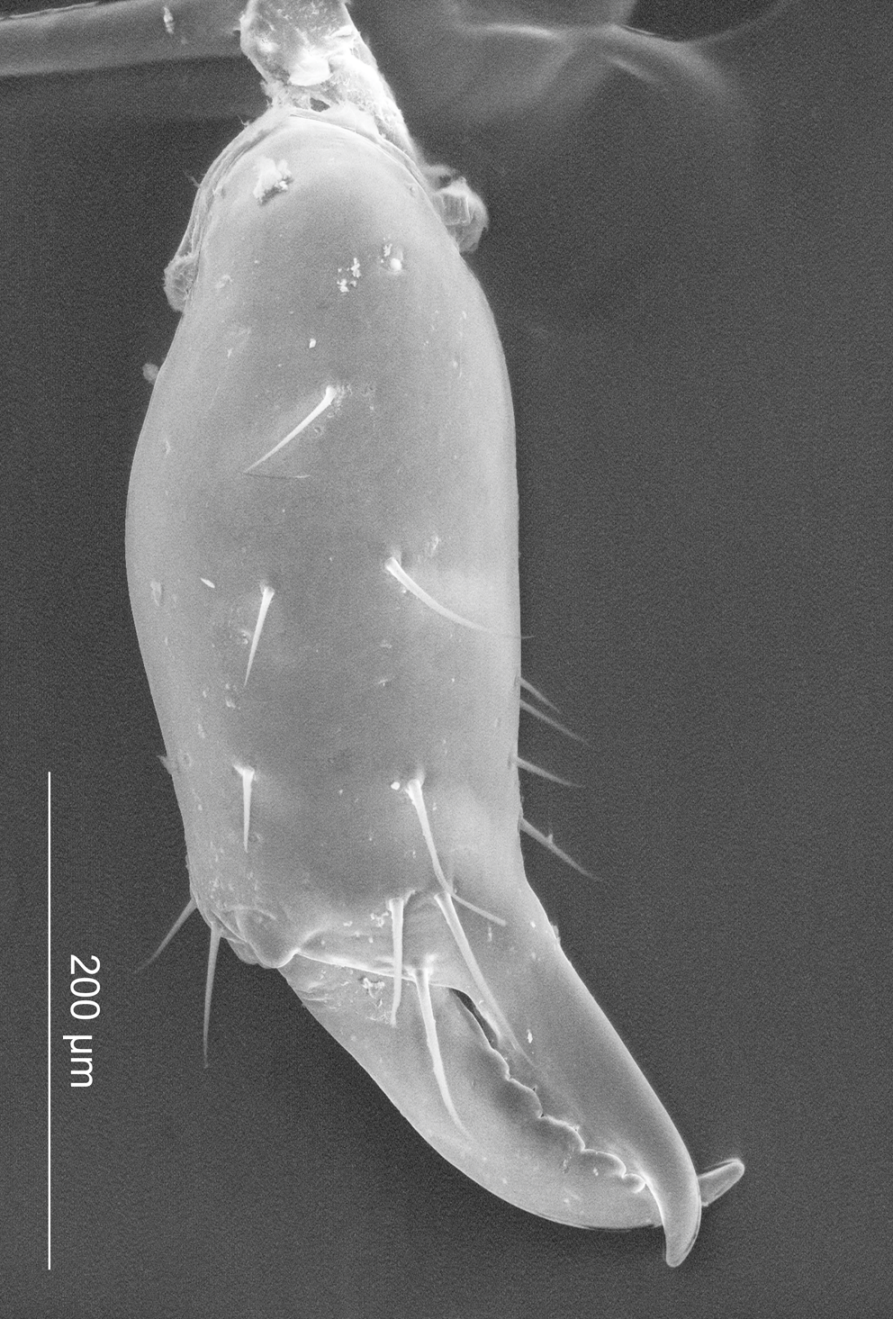
*Relictopiolus galadriel* gen. nov., sp. nov. male paratype (LES/UFSCar 0011189). Right cheliceral hand, frontal view.

**Fig 10:**
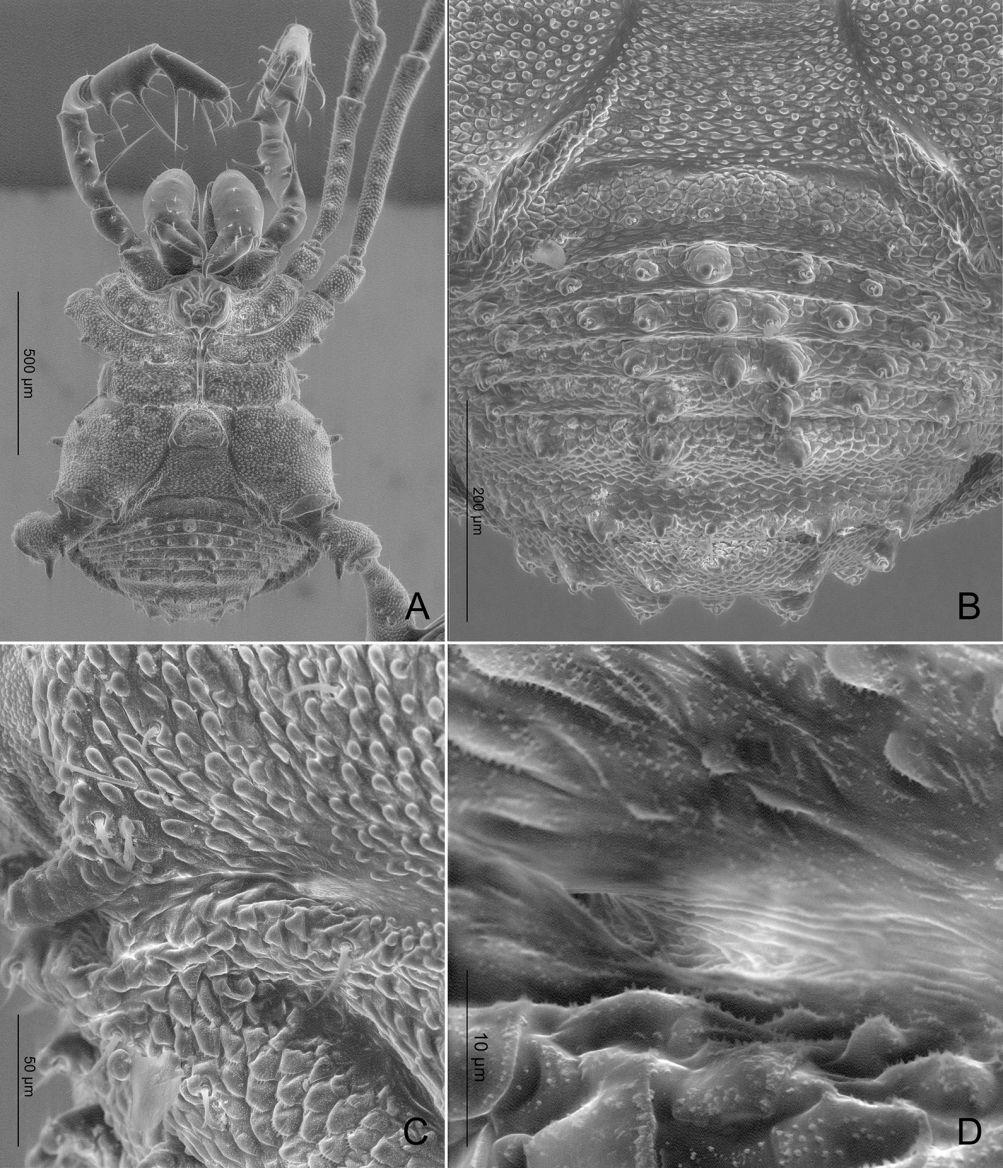
*Relictopiolus galadriel* gen. nov., sp. nov. male paratype (LES/UFSCar 0011189), ventral view. (A) Habitus. (B) Detail of free sternites and anal operculum. (C) Detail of spiracle area on right side. (D) Detail of concealed spiracle on right side. All images are oriented such that the anterior of the animal is at the top.

**Fig 11:**
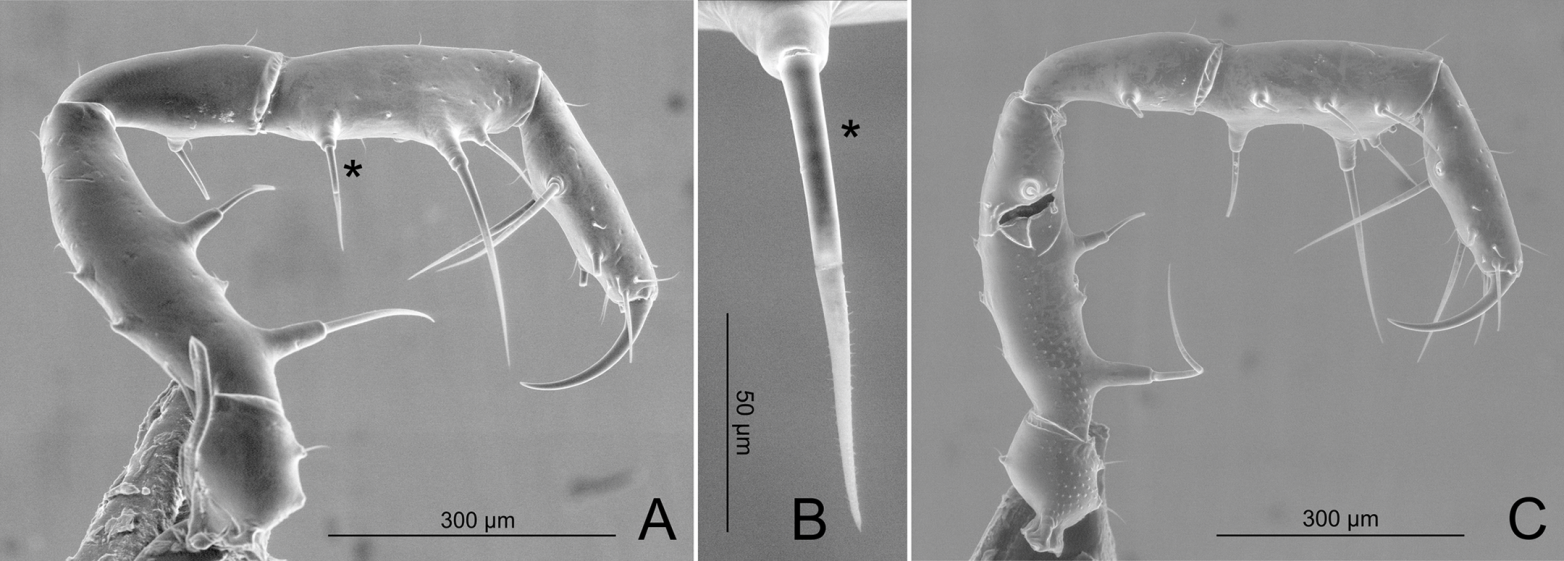
*Relictopiolus galadriel* gen. nov., sp. nov. male paratype (LES/UFSCar 0011189). (A) Right pedipalp, ectal view. (B) Detail of major spine indicated by *. (C) Left pedipalp, mesal view. *: major spine.

**Fig 12:**
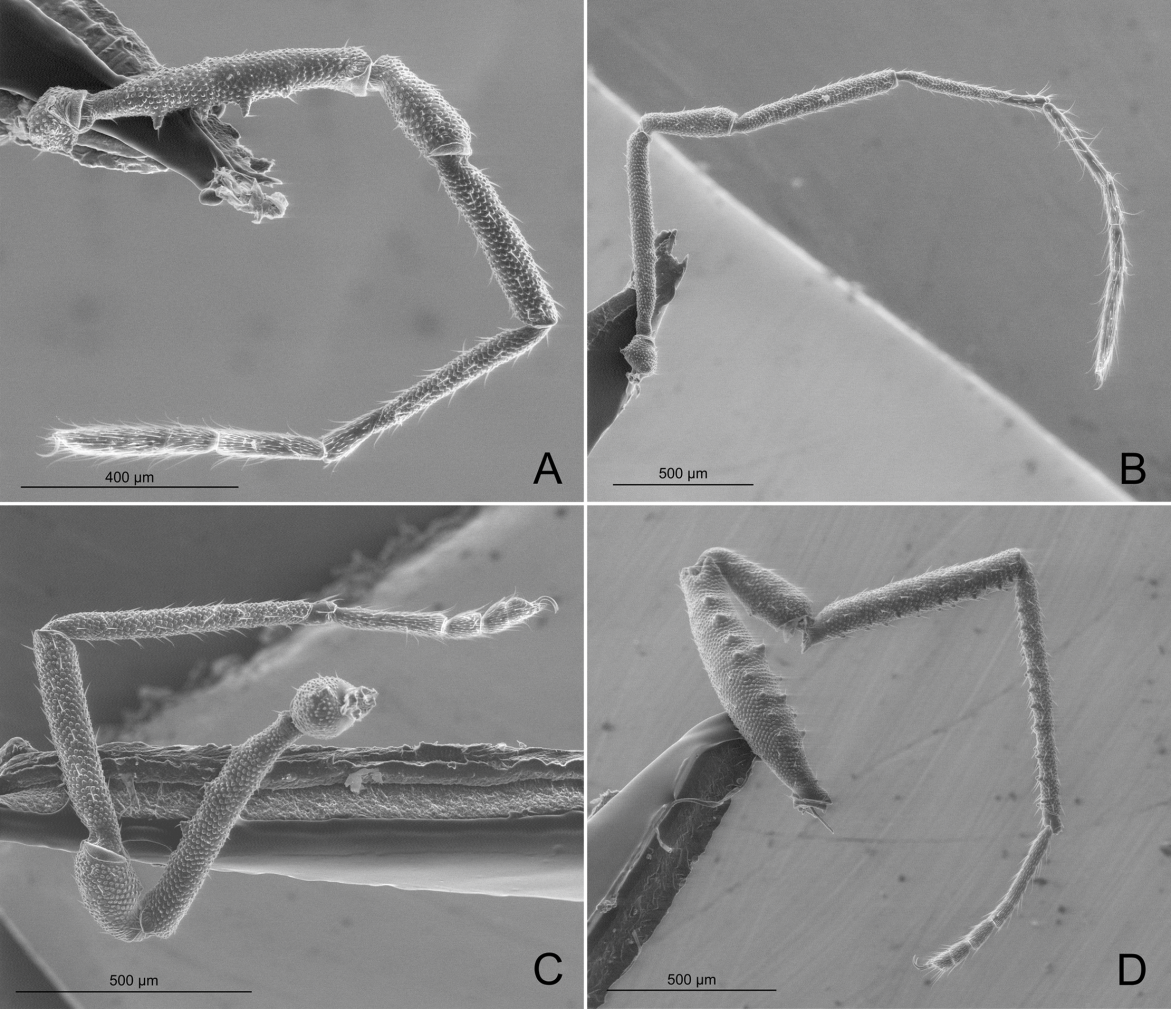
*Relictopiolus galadriel* gen. nov., sp. nov. male paratype (LES/UFSCar 0011189), right legs, retrolateral views. (A) Leg I. (B) Leg II. (C) Leg III. (D) Leg IV.

**Fig 13:**
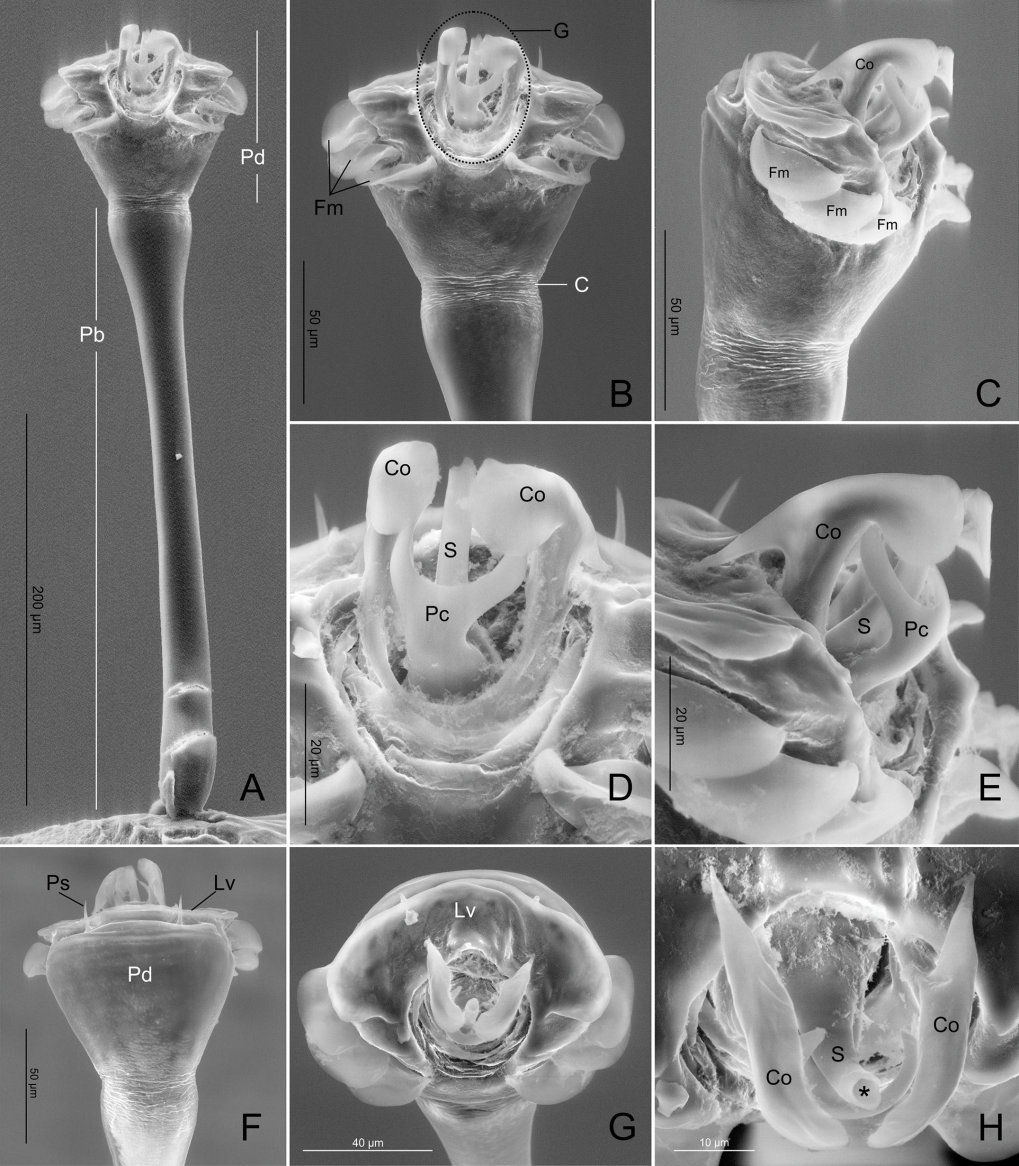
*Relictopiolus galadriel* gen. nov., sp. nov. male paratype (LES/UFSCar 0011189), male genitalia. (A) Dorsal view. (B) Detail of pars distalis, dorsal view. (C) Detail of pars distalis, lateral view. (D) Detail of glans, dorsal view. (E) Detail of glans, lateral view. (F) Detail of pars distalis, ventral view. (G) Detail of pars distalis, apical view. (H) Detail of glans, apical view. Abbreviations, C: constriction; Co: conductor; G: glans; Fm: flattened macrosetae; Lv: lamina ventralis; Pc: parastylar collar; Pb: pars basalis; Pd: pars distalis; Ps: pointed setae; S: stylus. *: ductus ejaculatorius opening.

**Fig 14:**
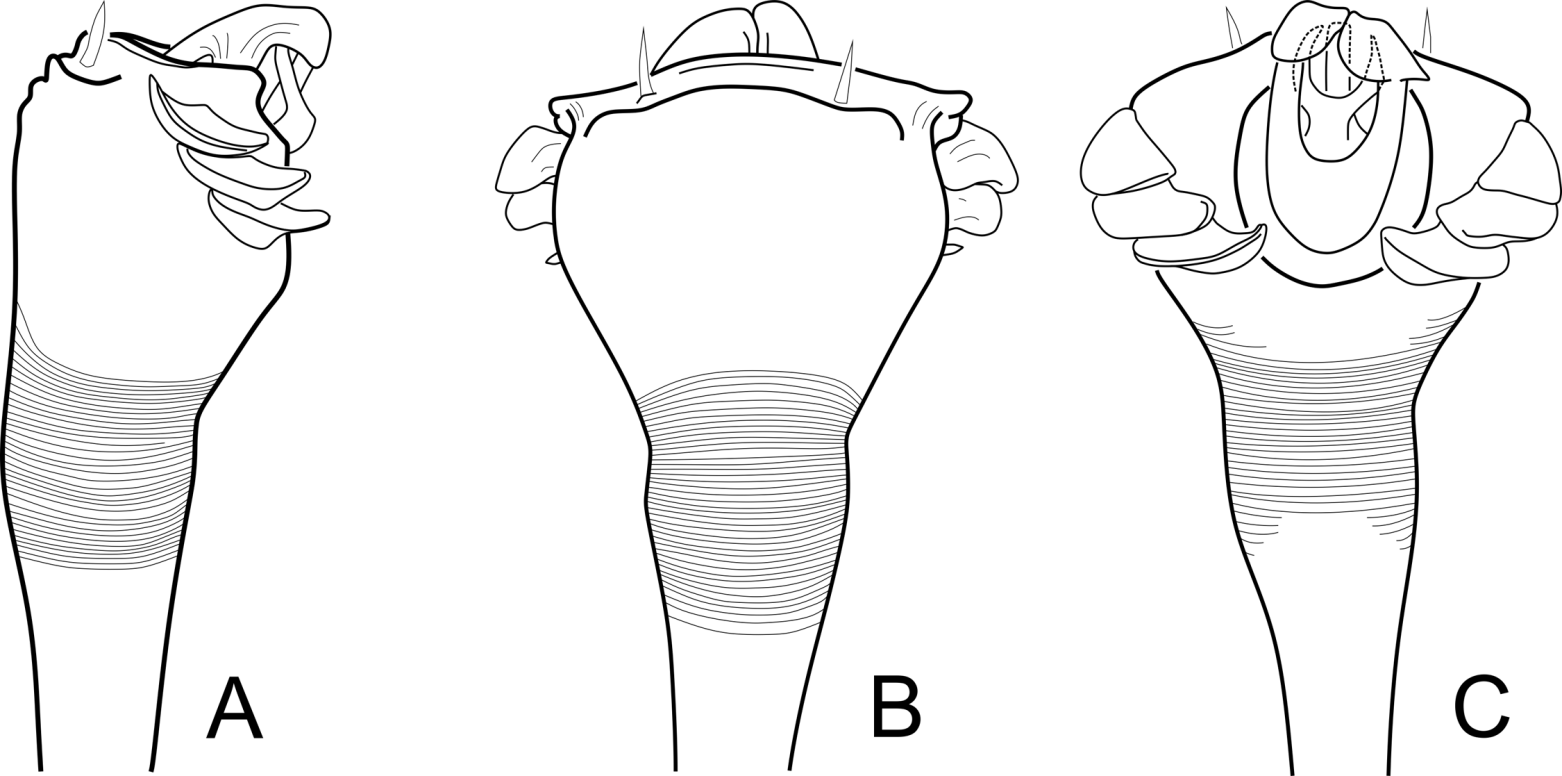
Line drawings of male genitalia of *Relictopiolus galadriel* gen. nov., sp. nov. male paratype (LES/UFSCar 0011189). (A) Detail of pars distalis, lateral view. (B) Same, ventral view. (C) Same, dorsal view.

**Fig 15:**
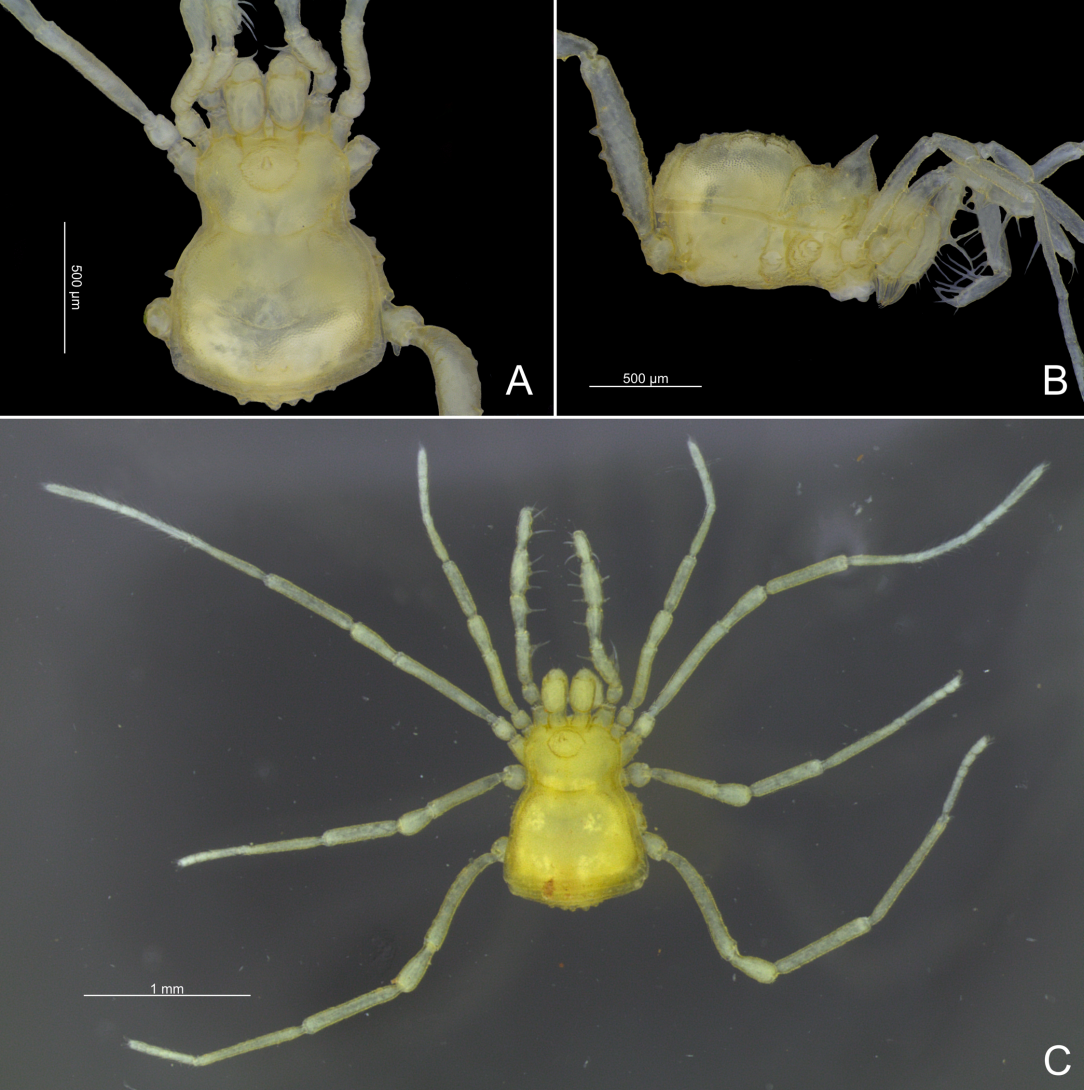
Habitus of specimens (in alcohol) of *Relictopiolus galadriel* gen. nov., sp. nov. (A) Male holotype (LES/UFSCar 0011188), dorsal view. (B) Same, lateral view. (C) Female paratype (LES/UFSCar 0011187), dorsal view.

**Fig 16:**
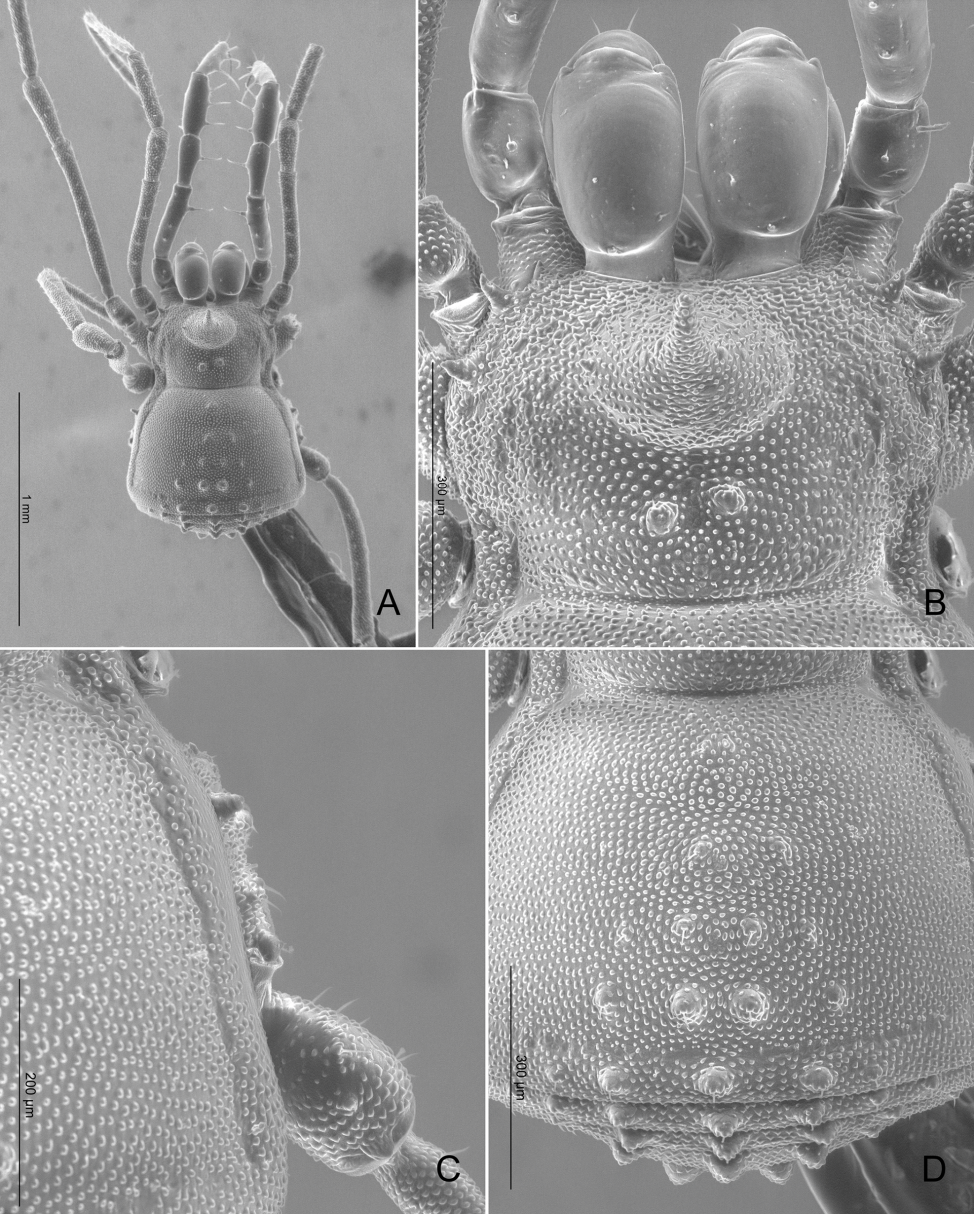
*Relictopiolus galadriel* gen. nov., sp. nov., female paratype (LES/UFSCar 0011190), dorsal view. (A) Habitus. (B) Detail of carapace and chelicerae. (C) Detail of right coxa IV. (D) Detail of mesotergal scute.

**Fig 17:**
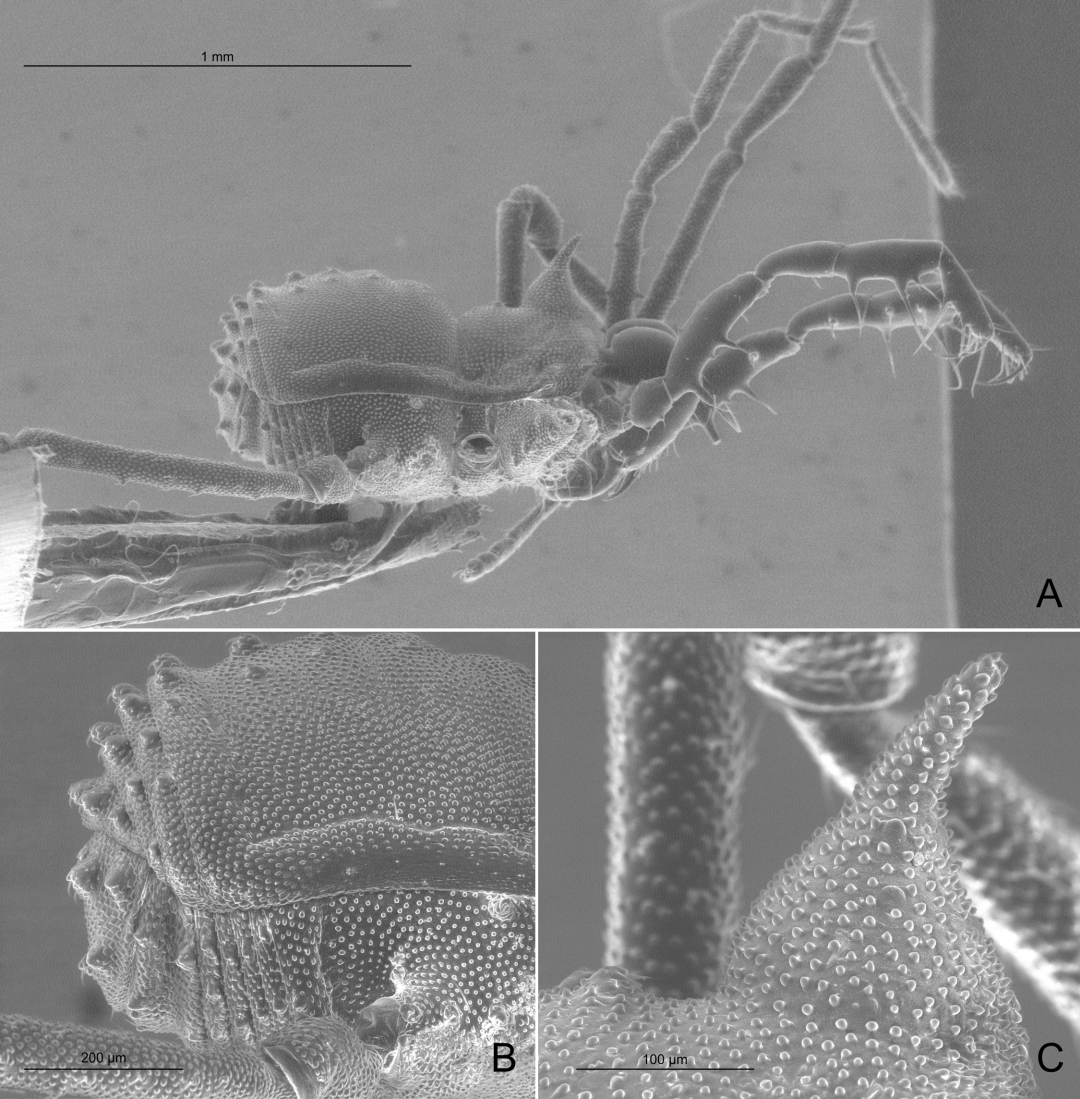
*Relictopiolus galadriel* gen. nov., sp. nov., female paratype (LES/UFSCar 0011190), lateral view. (A) Habitus. (B) Detail of posterior opisthosoma. (C) Detail of ocularium.

**Type material.** One male holotype (LES/UFSCar 0011188) from Olhos d’Água cave [15° 6'49.32"S, 44°10'10.56"W], Peruaçu Caves National Park, Itacarambi, Minas Gerais State, Brazil, 24.x.2015, Monte, B.G.O., Zepon, T.; three female paratypes (LES/UFSCar 0011187), same data as holotype; one male paratype (LES/UFSCar 0011189, SEM voucher), same data as holotype; one female paratype (LES/UFSCar 0011190, SEM voucher), same data as holotype; one female paratype (MZUSP), same data as holotype; one male paratype (MACN) from Olhos d’ Água cave [15° 6'49.32"S, 44°10'10.56"W], Peruaçu Caves National Park, Itacarambi, Minas Gerais State, Brazil, 26.viii.2014, Monte, B.G.O., Bolfarini, M.P.

**Etymology.** The species epithet is used as a noun in apposition. It refers to Galadriel (known as the Lady of Light), a character from J.R.R. Tolkien’s famous novel "The Lord of the Rings". This name was chosen to signify the importance of such a species which sheds light on the poorly understood evolutionary and biogeographic history of Kimulidae, particularly their ancestral distribution in South America.

**Diagnosis.** See diagnosis of the genus.

**Fig 18:**
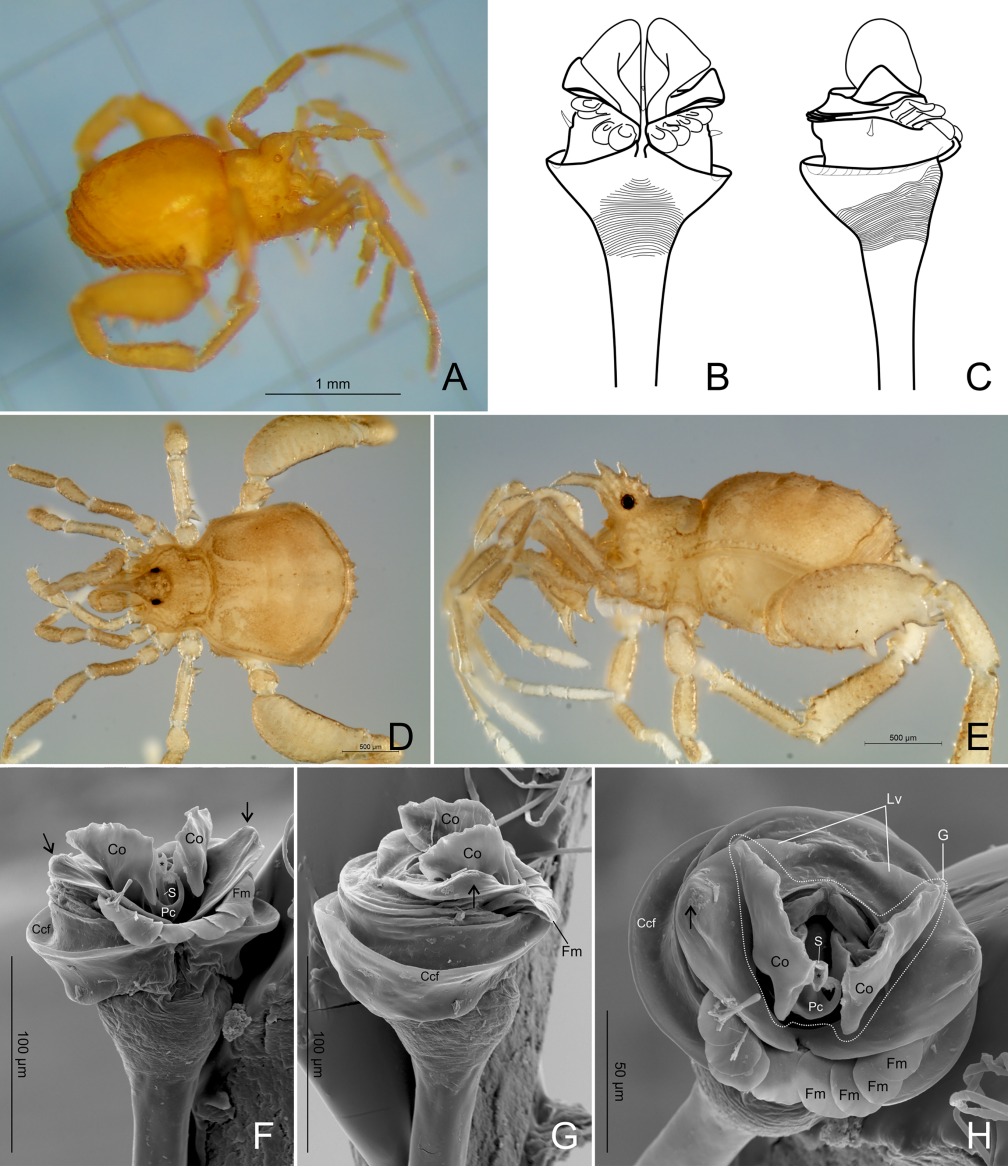
***Tegipiolus pachypus* Roewer, 1949, (A) male holotype and (B,C) male paratype (SMF 9906896, Tegipio, Pernambuco State, Brazil), (D-H) male (IBSP 7071, Murici, Alagoas State, Brazil).** (A) Habitus, dorso-lateral view. (B) Penis, dorsal view. (C) same, lateral view. (D) Habitus, dorsal view. (E) same, lateral view. (F) Penis, dorsal view. (G) Same, lateral view. (H) Same, apical view. Abbreviations, Ccf: circumplenial concave fold; Co: conductor; Fm: flattened macrosetae; G: glans; Lv: lamina ventralis; Pc: parastylar collar; S: stylus; *: ductus ejaculatorius opening; black arrows indicate the strongly convex lateral portion of lamina ventralis.

**Description.** Males (Figs 7–14,15A and 15B). Body measurements in Table 5. Entire body finely granulated (e.g. Fig 7). **Dorsum:** *scutum magnum*, bell-shaped with the mesotergal areas of approximately the same width, but slightly wider at the level of areas IV–V. Posterior margin of the *scutum* slightly convex. Carapace relatively wide compared to mesotergum (Ratio of mesotergum maximum width to carapace maximum width = 1.33). Carapace finely granulated, with the anterior margin slightly concave, cheliceral sockets not marked (Fig 7). Carapace in lateral view with a posterior ocularium region convex and armed with two obtuse granulated setiferous tubercles (Figs 7B and 8A), sulcus I well-marked. Antero-lateral border of the carapace armed with three acuminate strong tubercles (Fig 7B). Massive ocularium, granulated, terminating in a straight spiniform apophysis pointing anteriorly and with a broad and thick base (Fig 8B), and triangular in frontal view (Fig 8E). Eyes vestigial, apparently lacking the retina and cornea, with varying degree of degeneration, sometimes modified into a pointed tubercle (Figs 7B and 8B). Mesotergal scutum with five strongly convex areas (Figs 7D and 8A). Area I longer (along anterior-posterior axis) than remaining areas. Sulci between mesotergal areas I–IV incomplete, with a shallow, widened U-shaped line in sulci II and III (Fig 7D). Area I with a median region slightly elevated, covered with several low setiferous tubercles. Area II–IV with a median pair of low setiferous tubercles, larger on area IV. Area V with a transverse row of three low setiferous tubercles equal in size to those on area IV (Fig 7A and 7D). Free tergites each with one transverse row of low setiferous tubercles (Figs 7A, 7D, 8A and 8C). Ozopore region with well-marked descending, vertical and lateral channels (sensu Gnaspini and Rodrigues [62]) (Fig 8D). Coxa IV barely visible in dorsal view, terminating adjacent to sulcus III, with a prominent granulated obtuse setiferous tubercle at the prolatero-dorsal surface, near the mesotergal *scutum* border (Fig 7A and 7C). **Venter:** free sternites each with a transverse row of prominent acute setiferous tubercles, larger medially (Fig 10A and 10B). Anal operculum covered by many low, robust setiferous tubercles of the same size as those of the free tergites (Figs 8C and 10B). Coxa IV somewhat rounded, almost as wide as long, with several low wide setiferous tubercles (Fig 10A). Spiracles mostly concealed by coxa IV (Fig 10C and 10D). **Epistome:** epistome with sulcus well marked. Post-sulcal epistome wider than tall with a medial groove dividing the post-sulcal epistome into two convexes domes. Basal pre-sulcal epistome wide and short, almost triangular. Pre-sulcal epistome process long and triangular without median constriction (Fig 8F). **Chelicera:** basichelicerite unarmed with a well-marked rounded bulla. Cheliceral hand unarmed, normal, neither swollen nor hypertelic, covered with several sensilla. Mobile finger with uniform rounded teeth (Fig 9). **Pedipalp:** raptorial morphotype (sensu Wolff et al. [63]) (Fig 11A and 11C). Coxa short, unarmed, finely granulated. Trochanter globular, with one dorsal and two ventral small setiferous tubercle. Femur armed ventrally with one proximal and one medial major spines (i.e. stiff pointed bristles in highly elevated sockets, sensu Wolff et al. [63]) with one small pointed setiferous tubercle; dorsally with three small pointed setiferous tubercles and one subdistal-mesal major spine. Patella cylindrical, armed with one ventro-medial major spine on the mesal surface. Tibia armed ventrally with three ectal and three mesal major spines. Tarsus armed ventrally with three ectal and three mesal major spines (Fig 11A and 11C). All major spines possess very small and sparse microtrichia covering the distal half (Fig 11B). **Legs (Fig 12):** leg measurements in Table 5. Cuticle of legs is scale-like and granulated except on calcaneus and tarsus. Calcaneus restricted to the distal portion of legs. Femur I with a ventral row of four marked setiferous tubercles. Leg IV sexually dimorphic, males with a strong pointed tubercle in the ventro-distal portion of the trochanter (Fig 10A) and a moderately swollen femur with two ventral rows of low setiferous tubercles (Fig 12D). **Tarsal formula:** 3(2):4(2):4:5. **Male genitalia (Figs 13 and 14):** Pars distalis swollen separated from the pars basalis by slight constriction. Pars distalis with the lamina ventralis flat and slender, partially surrounding the glans, latero-dorsally with three pairs of huge, wide and flattened macrosetae and ventrally with two small pointed setae. Glans with a pair of laminar conductors expanded apically (hammer-like) and a wide stylus surrounded by a thin ring-like parastylar collar. **Remark:** the general groundplan of male genitalia resemble those of the close relative *Tegipiolus pachypus* (Fig 18B, 18C and 18F–18H), but exibits clear differences in the pars distalis (e.g. lamina ventralis, macrosetae and conductors morphologies). Furthermore, the external morphology of *Relictopiolus galadriel* is most similar to that of *Tegipiolus pachypus* (Fig 18A, 18D and 18E) compared to other kimulids. See *Relictopiolus* "comparative diagnosis" above for details.

**Table 5:** Somatic and appendicular measurements for *Relictopiolus galadriel* gen. nov. *et* sp. nov.

*Relicitopiolus galadriel* gen. nov. *et* sp. nov.		Male holotype (LES/UFSCar 0011188)	Male paratype (MACN)	Female paratype (LES/UFSCar 0011187)	Female paratype (LES/UFSCar 0011187)	Female paratype (LES/UFSCar 0011187)	Female paratype (MZUSP)
total body length		1.10	1.10	1.10	1.04	1.11	1.06
*scutum magnum* length		1.00	0.90	1.00	0.93	0.96	0.94
carapace length		0.40	0.40	0.40	0.38	0.39	0.38
carapace maximum width		0.60	0.60	0.60	0.55	0.55	0.63
mesotergal scute maximum width		0.80	0.80	0.80	0.81	0.83	0.86
	tr	0.10	0.10	0.10	0.10	0.12	0.09
	fe	0.40	0.40	0.40	0.35	0.38	0.39
Pedipalp	pa	0.20	0.20	0.20	0.17	0.20	0.19
	ti	0.30	0.30	0.30	0.25	0.30	0.26
	ta	0.30	0.20	0.20	0.23	0.25	0.24
	**Total**	**5.20**	**5.00**	**5.10**	**4.82**	**5.09**	**5.05**
	tr	0.10	0.10	0.10	0.09	0.10	0.10
	fe	0.50	0.50	0.50	0.50	0.49	0.51
	pa	0.20	0.20	0.20	0.22	0.26	0.24
Leg I	ti	0.30	0.40	0.30	0.32	0.34	0.33
	me	0.40	0.50	0.40	0.39	0.46	0.43
	ta	0.50	0.50	0.50	0.41	0.44	0.43
	**Total**	**2.00**	**2.20**	**2.00**	**1.92**	**2.10**	**2.05**
	tr	0.10	0.20	0.10	0.10	0.12	0.13
	fe	0.70	0.60	0.60	0.68	0.74	0.72
	pa	0.30	0.20	0.30	0.32	0.33	0.31
Leg II	ti	0.60	0.50	0.60	0.56	0.60	0.56
	me	0.50	0.60	0.50	0.45	0.50	0.50
	ta	1.00	0.50	1.00	0.96	1.05	1.00
	**Total**	**3.20**	**2.60**	**3.10**	**3.07**	**3.34**	**3.21**
	tr	0.10	0.20	0.10	0.12	0.10	0.09
	fe	0.60	0.60	0.60	0.56	0.59	0.54
	pa	0.20	0.20	0.20	0.19	0.20	0.21
Leg III	ti	0.50	0.50	0.40	0.40	0.47	0.41
	me	0.60	0.60	0.60	0.55	0.61	0.54
	ta	0.50	0.40	0.40	0.40	0.45	0.38
	**Total**	**2.50**	**2.50**	**2.30**	**2.22**	**2.42**	**2.17**
	tr	0.20	0.20	0.10	0.11	0.15	0.14
	fe	0.80	0.80	0.70	0.72	0.73	0.71
	pa	0.40	0.30	0.30	0.29	0.33	0.28
leg IV	ti	0.50	0.70	0.60	0.59	0.62	0.59
	me	0.80	0.80	0.80	0.71	0.78	0.72
	ta	0.50	0.60	0.50	0.50	0.55	0.50
	**Total**	**3.20**	**3.40**	**3.00**	**2.92**	**3.16**	**2.95**

Abbreviations: tr = trochanter, fe = femur, pa = patella, ti = tibia, ta = tarsus.

**Females (Figs 15C; 16 and 17).** Body measurements in Table 5. Similar in appearance to the males but without the swollen femur IV and with more tubercles in mesotergal areas (Fig 16).

**Color (in alcohol).** The color (both sexes) of the whole body is Brilliant Greenish Yellow (98). Legs are more transparent at apices, tarsus Pale Greenish Yellow (104), trochanter, femur, patella and tibia Light Greenish Yellow (101) (Fig 15).

**Distribution.** Known only from the type locality.

**Natural history.** Specimens were collected during five different expeditions to the Olhos d’Água cave, usually far from the entrance, about 1200 m from the resurgence (Fig 2A). Only one individual was collected nearer to the entrance, about 450 meters from the resurgence (Fig 2B). All individuals were observed and captured on the cave walls, in the largest galleries (3 m wide and 10 m high) (cave cross section I in Fig 2). Temperature (~28°C) and humidity (90% of RH) tend to be constant in the localities where the harvestmen were observed. Some individuals were observed in close proximity to two other troglobitic arachnids from Olhos d’Água cave: the opilionid *Iandumoema uai* Pinto-da-Rocha, 1997 and the amblypygid *Charinus eleonorae* Baptista and Giupponi, 2003. Other fauna observed in the galleries where *Relictopiolus galadriel* occurs includes nectarivorous and hematophagous bats, millipedes (Polydesmida), a troglobitic cricket (*Endecous peruassuensis* Bolfarini and Bichuette, 2015), coleopterans (families Cholevidae and Carabidae) and arachnids of the order Palpigradi.

#### Spurious Kimulidae: justification of generic exclusions and nomenclatural implications

*Acanthominua* Sørensen, 1932

*Acanthominua* [64]: 248; [65]: 91; [66]: 138; [67]: 40; [68]: 110; [69]: 62; [46]: 211 [type species: *Acanthominua tricarinata* Sørensen, 1932, by monotypy, (examined)].

*Phalangodinella* [70]: 5; [71]: 232; [46]: 249 [type specie: *Phalangodinella roeweri* Caporiacco, 1951, by monotypy, (not examined)]. **New synonymy.**

**Fig 19:**
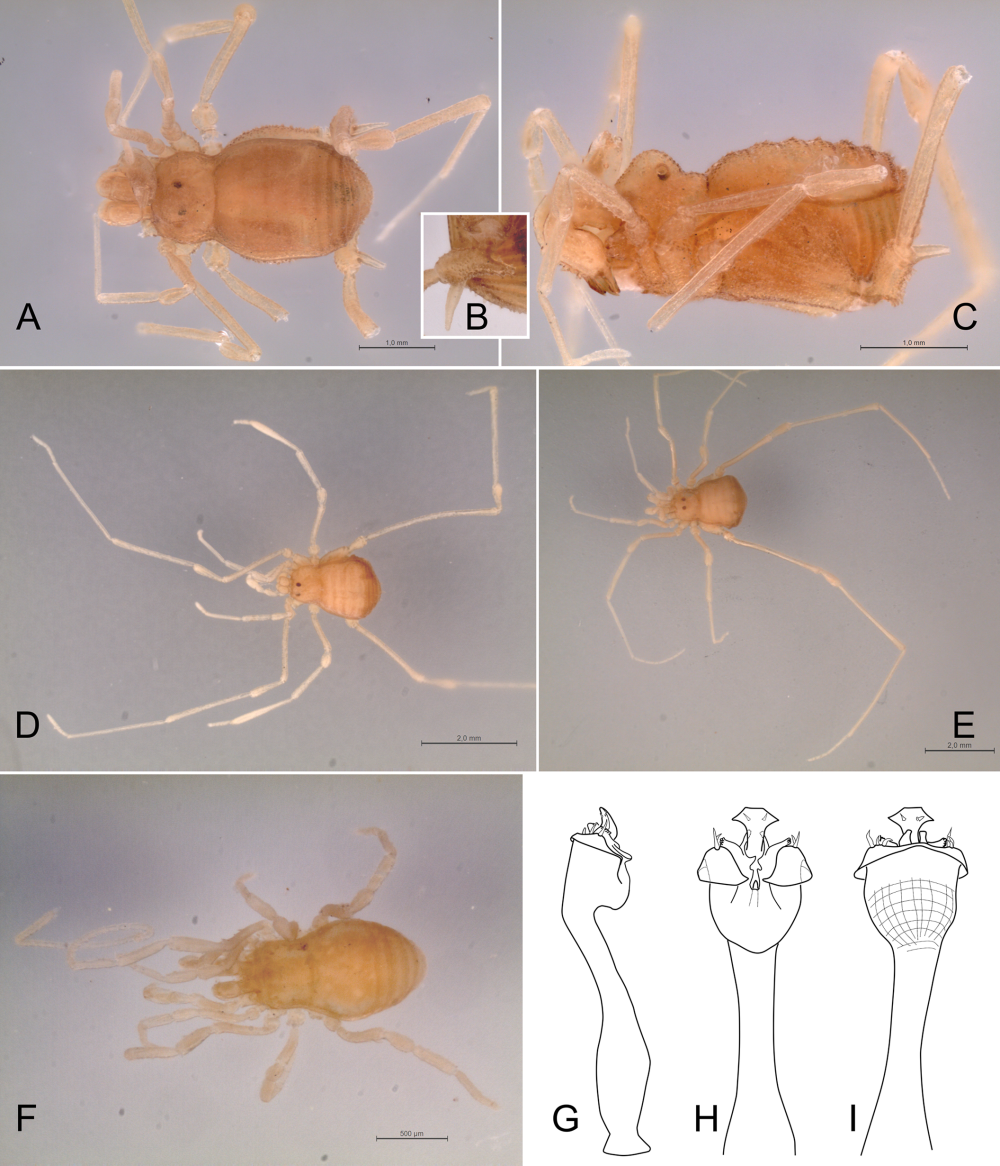
Spurious Kimulidae. (A‒C) *Acanthominua tricarinata* Sørensen, 1932 (male syntype, ZMUC). (A) Habitus, dorsal view. (B) Detail of right trochanter IV, ventral view. (C) Habitus, lateral view. (D) *Euminua brevitarsa* Sørensen, 1932 (male syntype, ZMUC), habitus, dorsal view. (E) *Euminua longitarsa* Sørensen, 1932 (male syntype, ZMUC), habitus, dorsal view. (F‒I) *Euminua convolvulus* Sørensen, 1932. (F) Habitus, dorsal view (male syntype, ZMUC). (G‒I) Male genitalia (male syntype, SMF). (G) Lateral view. (H) Dorsal view. (I) Ventral view.

The genus *Acanthominua* is currently considered a member of Kimulidae [61] but the examination of two males syntypes of the type species *Acanthominua tricarinata* Sørensen, 1932 (repository ZMUC) revealed that the exomorphology (Fig 19A–19C) does not match with the familial characteristics as defined by Pérez-González and Kury [72]; therefore, *Acanthominua* is herein transferred to Zalmoxidae Sørensen, 1886, **new family allocation**. Moreover, the morphology of one male syntype of *A*. *tricarinata* was highly congruent with the drawings of *Phalangodinella bicalcanei* Gonzalez-Sponga, 1987, including diagnostic characteristics such as the particular armature of trochanter IV with two huge apophyses (see [71]: 241, figs. 283–289 and Fig 19C). Type localities for these two species (Venezuela: Carabobo, Las Trincheras, for *Acanthominua tricarinata* and Venezuela: Carabobo, Puerto Cabello, San Esteban, 200 m. for *Phalangodinella bicalcanei*) are relatively close (~15 km). Therefore, we propose that *Acanthominua tricarinata* Sørensen, 1932 is a senior synonym of *Phalangodinella bicalcanei* Gonzalez-Sponga, 1987, **new synonymy**.

The genus *Phalangodinella* was established by Caporiacco [70] to accommodate the new species *Phalangodinella roeweri* Caporiacco, 1951. The species was described using a type series composed of four females and one juvenile from two different Venezuelan localities: El Junquito, D.F. and Rancho Grande (currently National Park Henri Pittier), Aragua. Gonzalez-Sponga [71] treated these as different species; he restricted *Phalangodinella roeweri* to the type locality (El Junquito) and described a new species, *Phalangodinella pittieri* González-Sponga, 1987, for the species living in Rancho Grande. González-Sponga [71] also described 11 more species under *Phalangodinella*. Comparing the descriptions and drawings of Caporiacco [70] and González-Sponga [71] we agree that the current 13 species comprising the genus *Phalangodinella* are congeneric based on the congruence of morphological and biogeographical data.

In his revisionary work, Gonzalez-Sponga [71] unfortunately did not examine any of Sørensen's types causing him to overlook the synonymy between *Acanthominua* and *Phalangodinella*. Following the priority principle [73] we consider *Acanthominua* Sørensen, 1932 as a senior synonym of *Phalangodinella* Caporiacco, 1951, **new synonymy**. The proposed synonymy results in the following new combinations: *Acanthominua araguitensis* (González-Sponga, 1987) **new combination**, *Acanthominua arida* (González-Sponga, 1987) **new combination**, *Acanthominua bicalcanei* (González-Sponga, 1987) **new combination**, *Acanthominua calcanei* (González-Sponga, 1987) **new combination**, *Acanthominua callositas* (González-Sponga, 1987) **new combination**, *Acanthominua caporiaccoi* (González-Sponga, 1987) **new combination**, *Acanthominua coffeicola* (González-Sponga, 1987) **new combination**, *Acanthominua longipes* (González-Sponga, 1987) **new combination**, *Acanthominua pilosa* (González-Sponga, 1987) **new combination**, *Acanthominua pittieri* (González-Sponga, 1987) **new combination**, *Acanthominua roeweri* (Caporiacco, 1951) **new combination**, *Acanthominua santaeroseae* (González-Sponga, 1987) **new combination**, *Acanthominua tropophyla* (González-Sponga, 1987) **new combination**.

*Additional remarks*: Kury [46] erroneously stated that the type locality for *Acanthominua tricarinata* was "Venezuela, Distrito Federal, Las Trincheras, (10°33'N, 66°59'W)". Sørensen [64] described the type locality as: "Patria: Venezuela. In the month of December 1891 at Las Trincheras CHR. LEVINSEN (LOFTING)…", and the original label contains the same data with a more specific date (21–23 of December of 1891). Gonzalez-Sponga [71] stated in his Historical Background that Sørensen, in 1891, worked on material collected by C. Levinsen in Las Trincheras, and determined this locality to be in the Carabobo State of Venezuela. Las Trincheras is a small town near the city of Valencia in the State of Carabobo (10°18'22.64"N, 68° 5'19.68"W, Google Earth).

*Euminua* Kury and Alonso-Zarazaga, 2011

*Euminua* [64]:239; [65]: 92; [66]: 138; [68]: 110; [69]:62; [71]: 205, [46]: 211. Unavailable name (see [74]:59).

*Euminua* [74]: 59 [type species: *Euminua brevitarsa* Sørensen, 1932, by original designation (examined)].

The genus *Euminua* Kury and Alonso-Zarazaga, 2011, was until now a member of Kimulidae [61]. The examination of three syntypes of the type species *Euminua brevitarsa* Sørensen, 1932, (repository ZMUC) revealed that the species exhibits a peculiar sexually dimorphic character: in males, leg IV is much longer than that of females (Fig 19D). This character is common in several genera and species of Zalmoxidae (e.g. [75]) several of which are recorded for Venezuela (e.g. *Ethobunus gracililongipes* (González-Sponga, 1987), *Chamaia convexa* González-Sponga, 1987, *Paraminuella bristowei* Caporiacco, 1951, *Traiania abundantis* González-Sponga, 1987 and *Unicornia flava* González-Sponga, 1987). Therefore, *Euminua* is herein transferred to Zalmoxidae Sørensen, 1886, **new family allocation**.

*Euminuoides* Mello-Leitão, 1935

*Euminuoides* [65]: 92; [66]: 138; [68]: 110; [46]:211 (type *Euminua longitarsa* Sørensen, 1932, by original designation, (examined)].

The genus *Euminuoides* Mello-Leitão, 1935 was, until now, a member of Kimulidae [61]. The examination of 14 syntypes of the type species *Euminua longitarsa* Sørensen, 1932, (repository ZMUC) revealed that the exomorphology (Fig 19E) does not match with the familiar characteristics as defined by Pérez-González and Kury [72] and the illustrations of the male genitalia by Sørensen ([64], Fig 5A–5C) show a form that is clearly related to other Zalmoxidae with a pergula and rutrum (as defined by Kury and Pérez-González [76]). Moreover, males of this species exhibit an elongated leg IV (Fig 19E), a sexually dimorphic character also described for *Euminua brevitarsa* (see above). Therefore, *Euminuoides* is herein transferred to Zalmoxidae Sørensen, 1886, **new family allocation**.

*Pseudominua* Mello-Leitão, 1933

*Pseudominua* [77]: 101; [66]: 138; [78]: 45; [71]: 205; [46]:213 [type species: *Euminua convolvulus* Sørensen, 1932, by original designation, (examined)].

The genus *Pseudominua* Mello-Leitão, 1933 was assigned to Kimulidae [61] but the examination of 5 syntypes of the type species *Euminua convolvulus* Sørensen, 1932, (ZMUC: 4 syntypes; SMF: 1 syntype) revealed that the exomorphology (Fig 19F) does not match with the familiar characteristics as defined by Pérez-González and Kury [72] and the male genitalia, with a pergula and rutrum, demonstrate their close relationship with Zalmoxidae (as defined by Kury and Pérez-González [76]) (Fig 19G–19I). Therefore, *Pseudominua* is herein transferred to Zalmoxidae Sørensen, 1886, **new family allocation**.

*Additional remarks*: Kury [46] erroneously stated that the type locality for *Pseudominua convolvulus* (Sørensen, 1932) was "Venezuela, Distrito Federal, Las Trincheras, (10°33'N, 66°59'W)". Sørensen [64] described the type locality as: "Patria: Venezuela. MEINERT collected 2 females and 5 males at Las Trincheras November 5th, 1891…", and the original label contains the same data. Gonzalez-Sponga [71] stated that the locality "Las Trincheras" is located in Carabobo State. Las Trincheras is a small town near the city of Valencia in the State of Carabobo (10°18'22.64"N, 68° 5'19.68"W, Google Earth).

### Implications for cave conservation

Some authors consider caves as hotspots of subterranean biodiversity if they contain 20 or more obligate subterranean species, a rather arbitrary number proposed by Culver and Sket [79]. Presently, there are 38 caves in the world that fall under this definition of a subterranean hotspot [79–81]). Taking a more comprehensive approach, Trajano et al. [82] adopted the concept of “spots of high diversity of troglobites” based not only on taxonomic richness, but also considering phylogenetic and genetic diversity. Thus, the Olhos d’Água cave, with at least 11 troglobites, as well as several other caves in Brazil, are considered important locales for subterranean diversity [82]. The Olhos d’Água cave contains taxa that represent phylogenetically and biogeographically important lineages, as well as genetic diversity, the latter based on the degree of specialization of individual species, that is, accumulation of autapomorphies. The presence of *Relictopiolus galadriel* in the Olhos d’Água is evidence that the cave provided a stable refugium for millions of years and acted as a "museum" of biodiversity. This indicates the need to consider some measure of phylogenetic and/or biogeographic relevance as additional criteria for ranking the importance of the different "spots of high diversity of troglobites" as defined by Trajano et al. [82]. In a separate study of this cave, data indicate that the Olhos d’Água cave is ecologically important based on its relatively high values of Taxonomic Distinctness (TD) (Monte and Bichuette pers. obs.).

The extraordinary diversity (i.e., morphological, phylogenetic, genetic and functional diversity) of the Olhos d’Água cave makes it a high conservation priority, but there are several challenges that must be overcome to ensure the continual protection of this fragile habitat and its unique biota. First, the cave’s resurgence, which serves as the only access point, is located outside of the PCNP boundaries, and therefore is not legally protected by the National Parks system in Brazil. Additionally, the protection of cave ecosystems, such as the Olhos d’Água cave, requires synergistic conservation efforts to protect connected ecosystems, including lotic and terrestrial ecosystems, which support life in the cave. The Olhos d’Água cave community is dependent upon allochthonous resources, or energy sources that are derived from outside of the cave, that are introduced to the cave by natural flash flooding in the region. Decreasing the input of organic matter can threaten the ecological functioning of a subterranean ecosystem by disrupting the trophic dynamics. Thus, flash floods are crucial to the maintenance of the cave community because they distribute organic matter 5000 m into the cave, thus providing energy sources that constitute a primary trophic level within the cave ecosystem. However, the number of flash flooding events has dramatically decreased over the last two decades, and this scarcity of floods has led to negative impacts on cave fauna, such as the significant decline in the population of the cave catfish *Trichomycterus itacarambiensis* Trajano and de Pinna, 1996 between 1994 and 2007 [83]. Another threat to the Olhos d’Água cave community is the increased use of groundwater for agricultural purposes in the region, accompanied by the construction of several dams upstream of the sinkhole and inside the cave to store water for use during the dry season (B.G.O.M and M.E.B., pers. obs.). Given that the Olhos d’Água cave system may be under intense ecological stress due to the scarcity of flash floods, the cavernicolous fauna is at risk of becoming endangered or extinct and conservation actions are urgently required in order to protect this delicate, valuable habitat and avoid causing irreversible damage to its unique biota.

*Relictopiolus galadriel* is the first relictual species discovered in the PCNP region and represents a key taxon for improving our understanding of the evolutionary and biogeographic history of an ancient lineage of harvestmen. Given that this troglobitic harvestman is a relictual species that exhibits low population density, and is endemic to a single cave that is under ecological stress due to global climate change as well as anthropogenic changes to the local environment, this species is a strong candidate for inclusion on the International Union for Conservation of Nature (IUCN) Red List of Threatened Species. Its inclusion on the Red List would establish a means for governmental authorities and the scientific community to make informed decisions and implement concrete conservation actions to prevent the loss of biodiversity in this cave.

## Supporting information

S1 Fig Fully-resolved phylogenetic tree obtained by Bayesian inference analysis of the complete concatenated dataset, conducted in MrBayes. Support values at nodes represent posterior probabilities.
(PDF)

S2 Fig Chronogram obtained by Bayesian inference analysis of the complete concatenated dataset, conducted in BEAST. Support values at nodes represent posterior probabilities and blue bars represent the 95% Highest Posterior Densities around divergence time estimates.
(PDF)

S3 Fig Fully-resolved phylogenetic tree obtained by Bayesian inference analysis of the complete concatenated dataset including COI and H3 third codon positions, conducted in MrBayes. Support values at nodes represent posterior probabilities.
(PDF)

S4 Fig Chronogram obtained by Bayesian inference analysis of the complete concatenated dataset including COI and H3 third codon positions, conducted in BEAST. Support values at nodes represent posterior probabilities and blue bars represent the 95% Highest Posterior Densities around divergence time estimates.
(PDF)
