# Supplementary figures and images for: Light from dark: A relictual troglobite reveals a broader ancestral distribution for kimulid harvestmen (Opiliones: Laniatores: Kimulidae) in South America
Source: PLoS One. 2017 Nov 30;12(11):e0187919. doi: 10.1371/journal.pone.0187919 (PMC5708626; doi:10.1371/journal.pone.0187919)

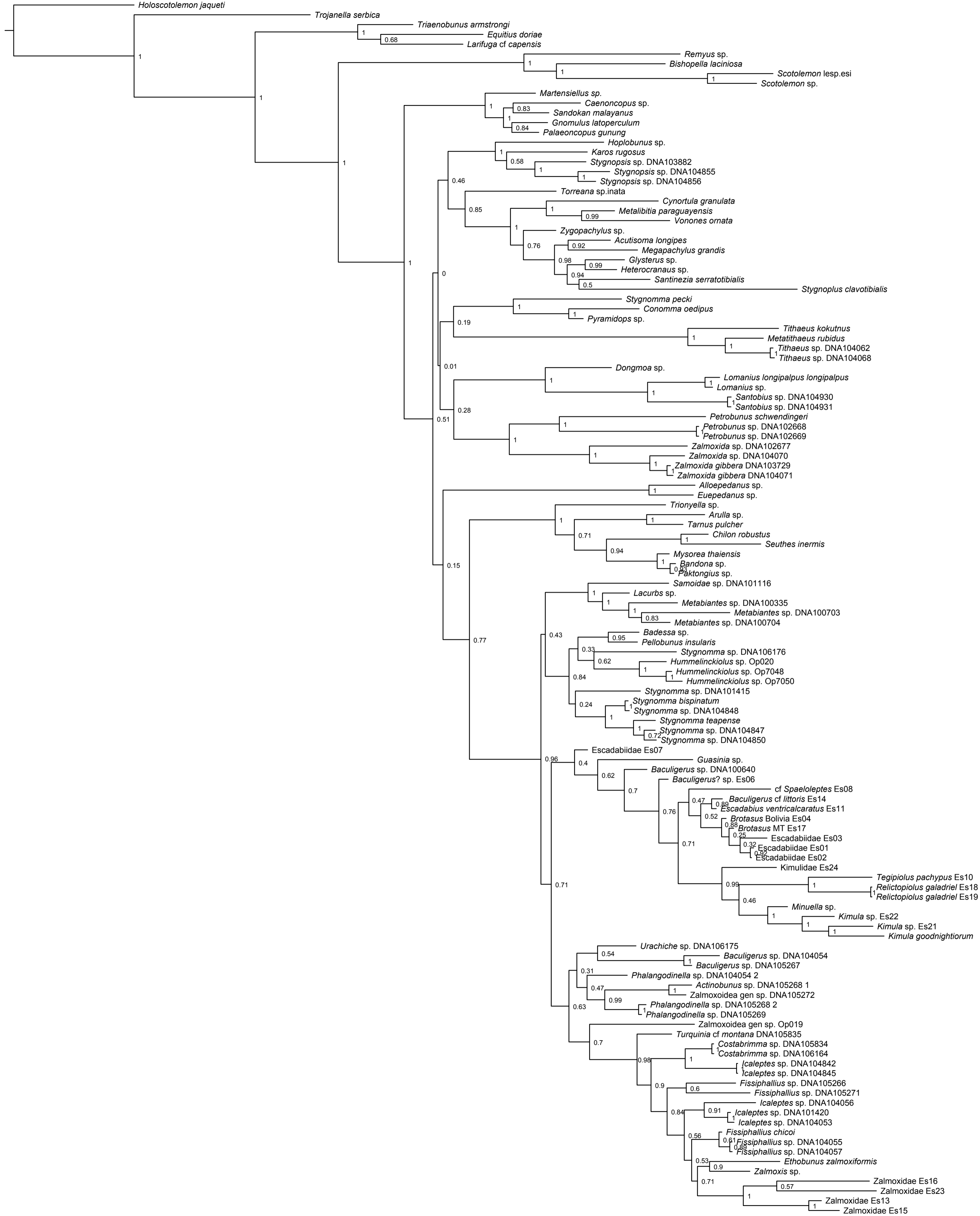

Supplement: S1 Fig — Support values at nodes represent posterior probabilities. (PDF) [file pone.0187919.s001.pdf]

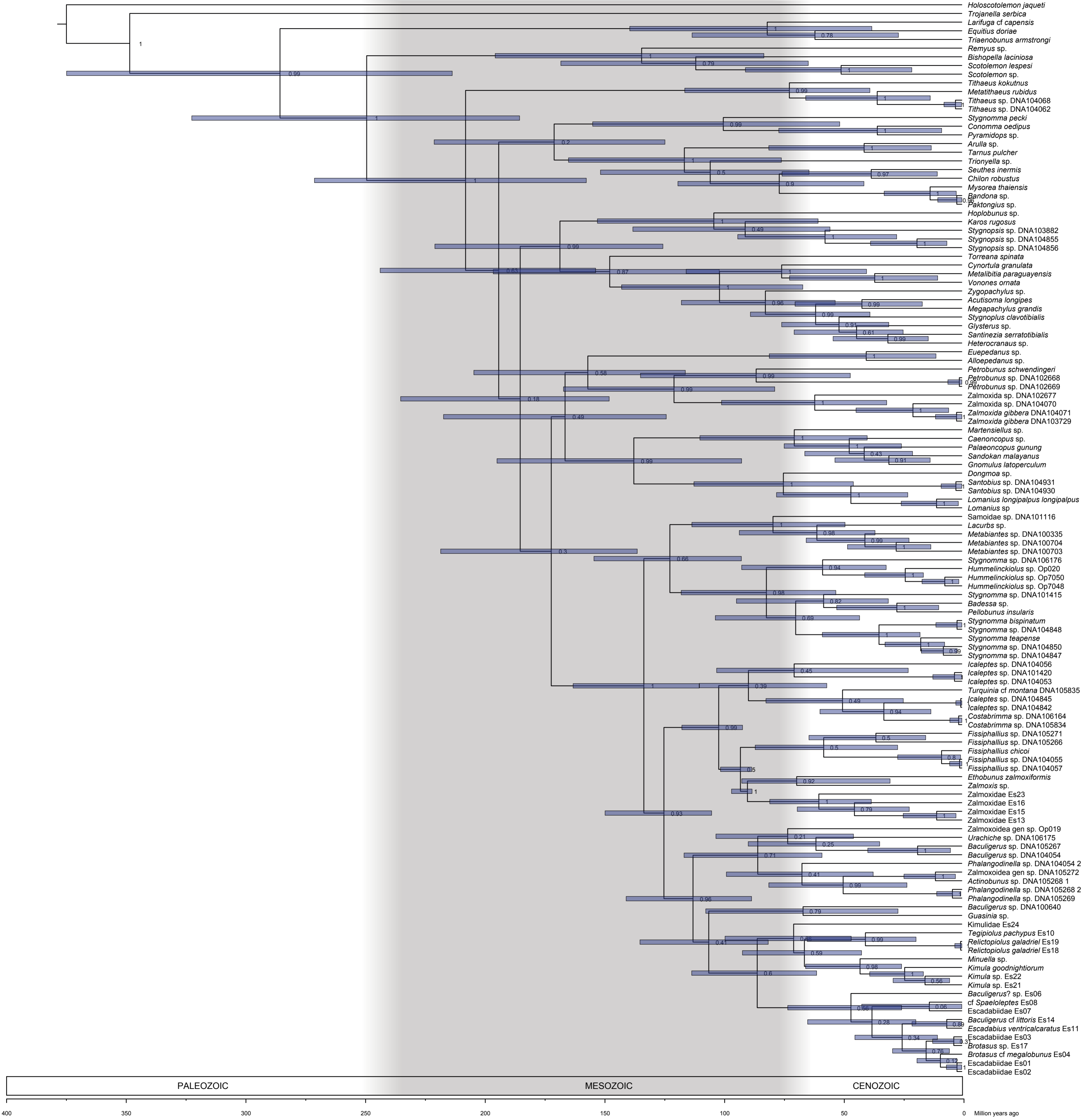

Supplement: S2 Fig — Support values at nodes represent posterior probabilities and blue bars represent the 95% Highest Posterior Densities around divergence time estimates. (PDF) [file pone.0187919.s002.pdf]

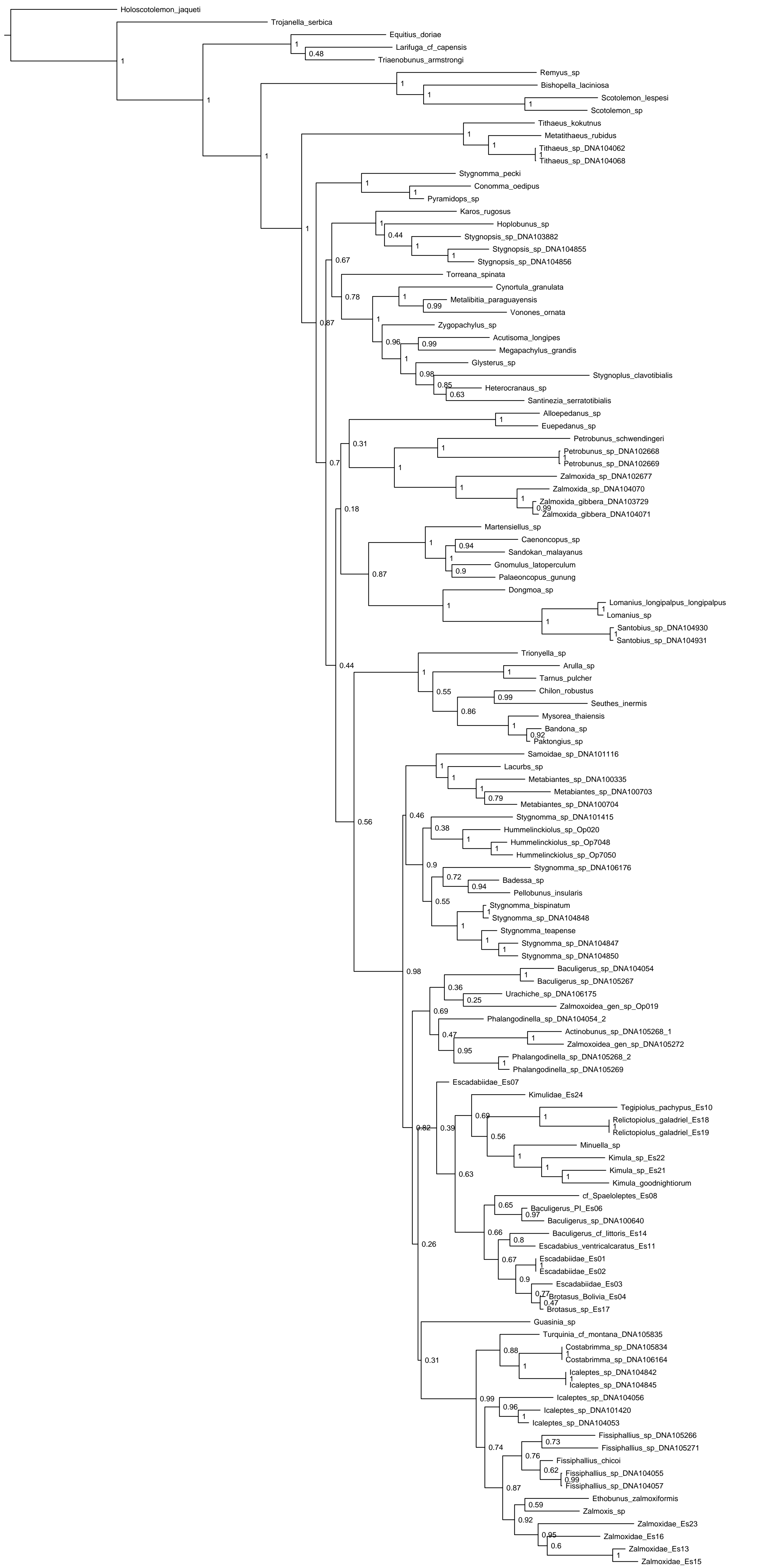

Supplement: S3 Fig — Support values at nodes represent posterior probabilities. (PDF) [file pone.0187919.s003.pdf]

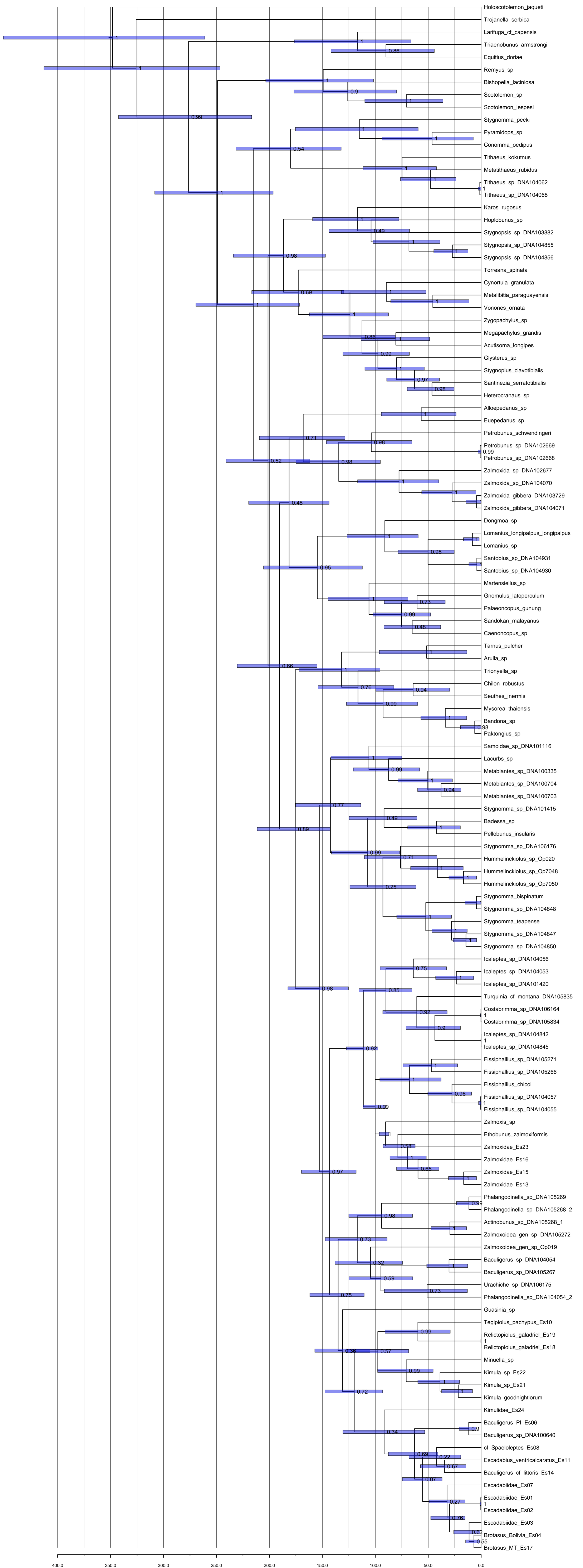

Supplement: S4 Fig — Support values at nodes represent posterior probabilities and blue bars represent the 95% Highest Posterior Densities around divergence time estimates. (PDF) [file pone.0187919.s004.pdf]
